# Histo-CADx: duo cascaded fusion stages for breast cancer diagnosis from histopathological images

**DOI:** 10.7717/peerj-cs.493

**Published:** 2021-04-27

**Authors:** Omneya Attallah, Fatma Anwar, Nagia M. Ghanem, Mohamed A. Ismail

**Affiliations:** 1 Department of Electronics and Communications Engineering, College of Engineering and Technology, Arab Academy for Science, Technology, and Maritime Transport, Alexandria, Alexandria, Egypt; 2 Computer and Systems Engineering Department, Alexandria University, Alexandria, Egypt

**Keywords:** Breast cancer, Computer aided diagnosis, Feature fusion, Deep learning, Histopathological images

## Abstract

Breast cancer (BC) is one of the most common types of cancer that affects females worldwide. It may lead to irreversible complications and even death due to late diagnosis and treatment. The pathological analysis is considered the gold standard for BC detection, but it is a challenging task. Automatic diagnosis of BC could reduce death rates, by creating a computer aided diagnosis (CADx) system capable of accurately identifying BC at an early stage and decreasing the time consumed by pathologists during examinations. This paper proposes a novel CADx system named Histo-CADx for the automatic diagnosis of BC. Most related studies were based on individual deep learning methods. Also, studies did not examine the influence of fusing features from multiple CNNs and handcrafted features. In addition, related studies did not investigate the best combination of fused features that influence the performance of the CADx. Therefore, Histo-CADx is based on two stages of fusion. The first fusion stage involves the investigation of the impact of fusing several deep learning (DL) techniques with handcrafted feature extraction methods using the auto-encoder DL method. This stage also examines and searches for a suitable set of fused features that could improve the performance of Histo-CADx. The second fusion stage constructs a multiple classifier system (MCS) for fusing outputs from three classifiers, to further improve the accuracy of the proposed Histo-CADx. The performance of Histo-CADx is evaluated using two public datasets; specifically, the BreakHis and the ICIAR 2018 datasets. The results from the analysis of both datasets verified that the two fusion stages of Histo-CADx successfully improved the accuracy of the CADx compared to CADx constructed with individual features. Furthermore, using the auto-encoder for the fusion process has reduced the computation cost of the system. Moreover, the results after the two fusion stages confirmed that Histo-CADx is reliable and has the capacity of classifying BC more accurately compared to other latest studies. Consequently, it can be used by pathologists to help them in the accurate diagnosis of BC. In addition, it can decrease the time and effort needed by medical experts during the examination.

## Introduction

Cancer is considered to be one of the most life-threatening diseases affecting human health (Toğaçar et al., 2020). Based on World Health Organization (WHO) reports, cancer is currently the second reason for death worldwide and has led to the death of approximately 8.8 million individuals in 2015 (GBD 2013 Risk Factors Collaborators, 2015). Among the various types of cancer, today breast cancer (BC) is one of the most widely spread kinds of cancer among women in 140 of 184 countries globally (Torre et al., 2017). Annually, more than 1.5 million women are diagnosed with BC; 15% of the total number of deaths are due to BC (Bhuiyan et al., 2019; Anwar et al., 2020). This number is estimated to reach 19.3 million in 2025 according to the WHO (Ragab, Sharkas & Attallah, 2019). Therefore, early and accurate diagnosis of BC is of great importance to lower death rates, since almost 96% of cancers are treatable in primary phases (Ragab, Sharkas & Attallah, 2019).

The most common imaging modalities for the screening of BC are mammograms, MRI (Magnetic Resonance Imaging), ultrasound, and histopathology (Saxena & Gyanchandani, 2020). Among these modalities, histopathology is considered an excellent method for the diagnosis of cancer (Spanhol et al., 2015; Sharma & Mehra, 2020a; Saxena & Gyanchandani, 2020). In this technique, affected breast tissue is extracted and inspected under the microscope. Histopathology provides direct confirmation for classification, evaluation, and experimental management. Nevertheless, a histopathology slide has complex visual patterns that could be hard to be classified as benign or malignant (Arevalo, Cruz-Roa & Gonzalez, 2014). Moreover, pathological analysis is difficult, time-consuming, and is subjective to experts’ experience. Besides, professional pathologists may come up with inaccurate diagnosis (Zhang et al., 2019). Furthermore, the limited availability of pathologists is a severe obstacle in the analysis of histopathological images. There is only one pathologist for every 100,000 and 130,000 individuals in sub-Saharan Africa and China respectively (Wilson et al., 2018). Similarly, the number of available pathologists in India and the USA is one for every 65,000 individuals, and 5.7 for every 100,000 individuals respectively (Robboy et al., 2013). Therefore, this shortage in pathologists increases the burden on current pathologists. For these reasons, there is an essential need for automatic BC classification to reduce the burden exerted by the pathologist in identifying BC, and further assist them in conducting accurate BC diagnosis.

With the latest enhancement of machine learning and deep learning, approaches, CADx schemes have led to great improvements in the automatic diagnosis of BC in its early stages (Ragab et al., 2021). CADx can help doctors and pathologists in analyzing histopathological images, thus reducing the cost of diagnosis (Kumar et al., 2020). But such systems suffer from some challenges when analyzing histopathological images as these types of images contain complicated patterns and sophisticated textures. Moreover, choosing the appropriate feature extraction approach that has a strong capacity to extract significant features from such complicated patterns and textures (Xie et al., 2019). Feature extraction is the main step in the accurate diagnosis of medical conditions (Baraka et al., 2019) such as BC (Ragab, Sharkas & Attallah, 2019), gastrointestinal diseases (Khan et al., 2020; Ali et al., 2019; Khan et al., 2019; Attallah & Sharkas, 2021), heart diseases (Attallah et al., 2017a; Attallah & Ma, 2014; Karthikesalingam et al., 2015; Attallah & Ma, 2015), and brain disorders (Attallah, 2020; Ceschin et al., 2018; Attallah, Sharkas & Gadelkarim, 2019; Attallah, Gadelkarim & Sharkas, 2018). Accordingly, CADx systems are characterized into two categories. Traditional feature extraction methods are the first category. In such methods, prior knowledge of the nature of the data is needed to extract useful features (Attallah, 2019). These methods often require finding a reasonable trade-off between accuracy and computation efficiency (Nanni, Ghidoni & Brahnam, 2017). On the other hand, the deep learning (DL) methods are the other category. This category can extract features from images in an automatic manner (Zhang et al., 2019). Although such category has great capabilities for classifying and extracting features from huge datasets, DL is not always the perfect option in all datasets especially these having a small number of images (Nguyen et al., 2018; Ragab & Attallah, 2020). DL methods extract features automatically, thus they do not need guidance to perform the feature extraction procedure like traditional feature extraction approaches. However, these methods may suffer from overfitting with small training datasets (Zhang et al., 2017; Ragab & Attallah, 2020; Nanni, Ghidoni & Brahnam, 2017). Combining the previous two categories may be able to enhance the classification accuracy (Hansley, Segundo & Sarkar, 2018; Hasan et al., 2020; Wei et al., 2019; Ragab & Attallah, 2020).

We propose an automatic CADx system (Histo-CADx) to diagnose BC from histopathological images, which relies on combining the traditional feature extraction approach with multi-DL approaches. Various convolutional neural networks (CNN) are explored for feature extraction. Several spatial features pulled out from such CNNs models are merged by several significant texture-based feature extraction approaches using AE (auto-encoder) model. Details of the CNNs and AE are going to be illustrated in the methods section. Histo-CADx is based on two fusion stages. In the first stage, the system fuses multiple features extracted from different paradigms using a DL approach. Then, the system explores the best combination of feature fusion set that influence the performance of the CADx. In the second stage, the system fuses outputs from several machine learning classifiers to investigate the impact of combining several classifiers.

## Related Work

Several CADx systems have been proposed to diagnose BC using histopathological images. Many of these CADx systems used the BreakHis dataset (Spanhol et al., 2015). A CNN is merged with a boosted trees model for BC classification was introduced in Vo, Nguyen & Lee (2019). The system utilized ensembles of Inception-ResNet-v2 convolutional neural network (CNN) to extract spatial features which were then classified using a gradient boosting trees classifier and obtained an accuracy of 99.5%. An Inception module-based CNN was also used in Burcak, Baykan & Uğuz (2020). Toğaçar et al. (2020) designed a novel CNN architecture named BreastNet for BC diagnosis. The system is based on only one network, spatial features, and only one dataset. Xie et al. (2019) used the Inception V3 and Inception ResNet V2 CNNs to extract deep features and construct their classification model. The authors found that the features of the Inception-ResNet V2 are more powerful than the Inception V3. Thus, they used the Inception-ResNet V2 to perform more analysis using k means clustering. Then, they employed an AE to reduce the dimension of features. Finally, they used SVM and k-NN classifiers to perform classification. In the same year, Xu et al. (Xu et al., 2019) proposed a CADx system for classifying BC using three stages. The first stage used a hard-attention sensor to determine if a region is abnormal or not, then in the second stage, the authors used SA-Net to extract features from these regions. Finally, the authors used LSTM for classification. Yang et al. (2019) used three CNNs models including the DenseNet-161, ResNet-152, and ResNet-101 CNNs to classify BC. They indicated that the fusion of the three fine-tuned models was more accurate than individual CNNs. The proposed EMS-Net model showed an accuracy of 91.75%. This system was based only on spatial features, only one dataset, and yielded low accuracy. Budak et al. (2019) presented a CADx system based on features extracted from the AlexNet CNN and used it to train a bidirectional Long short-term memory (LSTM). This study yielded an accuracy of 98.10%. Similarly, Nahid & Kong (2018) extracted deep features from a CNN and used them to train an LSTM network to distinguish histopathology images of BC. In the same year, Nahid, Mehrabi & Kong (2018) extracted features from CNN and LSTM networks then performed clustering using k means clustering and mean shift clustering. Afterward, they used an SVM classifier for classification achieving an accuracy of 91%. In 2020, Gour, Jain & Sunil Kumar (2020) implemented a residual learning-based CNN called ResHist and attained an accuracy of 92.52%. Sharma & Mehra (2020b) proposed a new transfer learning method for the AlexNet CNN and attained an accuracy of 89.31%. The authors (Lin & Jeng, 2020) proposed a novel CNN that is optimized using the uniform experimental design method. Although the method was time-efficient, it achieved a low mean accuracy of 88.41%. Besides, this system used only one network for BC classification. Also, the authors used only spatial features. Jiang et al. (2019) introduced a novel CNN structure that has a convolutional layer, and a fully connected layer (FC), and a small SE-ResNet module, to analyze histopathology images, a 94.43% accuracy was obtained. Some of the previous systems used only one CNN to extract spatial features. Other studies did not examine the influence of feature fusion, On the other hand, the limitations of (Xie et al., 2019; Xu et al., 2019; Toğaçar et al., 2020; Burcak, Baykan & Uğuz, 2020) techniques are using only one type of CNN to extract features. Moreover, the authors extracted only spatial features to construct their model. The models were evaluated using only one dataset.

In Mehra (2018), a comparison study was performed to test the performance of the VGG-16, VGG-19, and ResNet-50 CNNs. A 92.6% accuracy was obtained by the VGG-16 CNN via regression compared to 79.40% and 90.0% of the ResNet50 and VGG-19 CNNs. In Nawaz, Sewissy & Soliman (2018), features taken out from three CNNs including (Inception-V1, ResNet-V1, and Inception-V2) are tested to determine which of them has better accuracy, the result of this comparison indicated that the ResNet CNN has the maximum classification accuracy of 95%. Later in Zhu et al. (2019), the authors proposed a hybrid model based on the Inception and residual modules combined with the Batch Normalization technique and attained an accuracy of 85.7%. Additionally, (Nahid & Kong, 2018) five CNN networks were built, taking together conventional and DL-based feature extraction approaches. This method achieved the maximum performance of 92.19%. The classification accuracies obtained by the previous three methods were not quite high. These methods used individual CNNs for feature extraction and classification. They did not explore the influence of feature fusion. They only used spatial information for classification. On the other hand, Dimitropoulos et al. (2017) suggested a model for the identification of BC by encoding histological images as representations of Vector of Locally Aggregated Descriptors (VLAD) on the Grassmann Manifold. In the same year, Wei et al. (2017) introduced two methods to classify BC. The first approach was based on extracting a collection of handcrafted features encoded by two coding models (word bag and locality restricted linear coding). Such features are used to train SVM classifiers. The second approach was based on the design of a novel CNN. The authors compared the performance of the handcrafted-based model and the CNN-based model and showed that the proposed CNN was superior to the manual feature-based classifier. The accuracy achieved was 97.50%. The weakness in such a technique was that the authors did not explore the effect of feature fusion of DL and handcrafted features, and only one CNN was used to extract features.

Lately, numerous CNN-based methods for automatic and accurate classification of BC pathological images were established for the ICIAR2018 challenge (Aresta et al., 2019). The core designs of these approaches are quite similar. Initially, images are preprocessed and then split into equal regions. Afterward, features are extracted using three CNNs. Vesal et al. (2018) proposed a CADx system based on the fusion of the outputs of the GoogleNet and ResNet CNNs using majority voting. Vang, Chen & Xie (2018) introduced a CADx system based on the Google Inception- V2 CNN to achieve a patch-wise classification. The process was made using an ensemble of gradient boosting machine and logistic regression. On the other hand, Rakhlin et al. (2018) presented a novel CNN model namely deep convolutional feature representation. The authors first used their CNN model for feature extraction. Then, they used gradient boosted trees as classifiers. Roy et al. (2019) proposed a CADx that extracted features using a CNN and used majority voting to take a final decision. Sitaula & Aryal (2020) proposed a method that extracts multiple types of features using VGG-16. These features include foreground and background information at two different feature extraction levels (low level of feature corresponding to background features and high level of features representing the foreground features). Sheikh, Lee & Cho (2020) proposed a multi-scale input and multi-feature network (MSI-MFNet) model, which fused features extracted from the network’s dense connectivity structure. The authors used two datasets to evaluate their model, namely the BreakHis and ICIAR2018 challenge datasets. They achieved an accuracy of 92% and 83% on the BreakHis and ICIAR 2018 datasets, respectively. All previously mentioned techniques were based on spatial features only. Some of them did not achieve high accuracy and models were evaluated on only one dataset. Most of them used only one CNN for feature extraction. They did not explore the fusion of several features extracted from multiple CNNs. Also, almost all of them did not investigate the influence of fusing features from multiple CNNs and handcrafted features. To the best of our knowledge, no study has examined the best combination of DL and handcrafted features that impact the performance of a CADx. Therefore, in this study, we designed a CADx system to address these limitations and present the following contributions.

- Proposing an automatic and accurate CADx system (Histo-CADx) which is based on duo cascaded fusion stages.
- Exploring various Deep CNN-based approaches for feature extraction instead of using one or two networks.
- The first fusion stage of Histo-CADx is a feature-based fusion process that employs a feature fusion strategy using an auto-encoder (AE) DL technique. During this stage, spatial features extracted from five CNNs networks are combined with the AE model along with several significant texture-based features extracted using traditional feature extraction approaches based on textural analyses.
- Proposing a feature set selection method that searches for the appropriate set of fused features among feature sets generated from the multiple CNNs and handcrafted features using sequential forward and backward search strategies.
- The second fusion stage of Histo-CADx includes the fusion of three classifiers. A multiple classifier system (MCS) is developed to classify BC, where the classification results of all classifiers are merged using a majority voting approach to obtain a more accurate diagnostic decision.
- Investigating if the process of fusing several spatial DL features with traditional feature extraction methods, and then selecting among these feature sets is capable of enhancing the performance of the introduced CADx.
- Generating a final model after the two feature fusion stages that can be considered as a reliable CADx and highly accurate compared to others available in the literature.
- Testing the performance of the model on two public datasets.

## Materials and Methods

### Dataset description

#### BreakHis dataset

**Figure 1:**
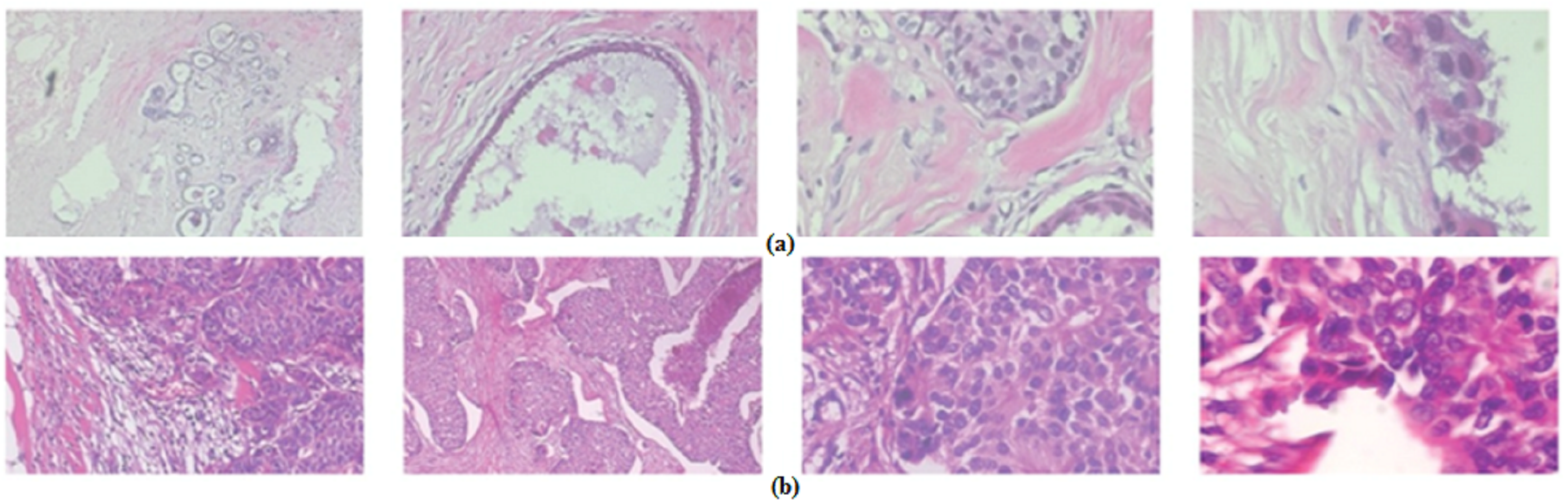
Samples of BreakHis dataset images with a 400× amplification factor: (A) malignant images, (B) benign images.

The BreaKHis dataset (Spanhol et al., 2015) is freely accessible and is commonly used for BC classification issues. This dataset contains 7,909 images of two primary classes: malignant or benign. The malignant subset contains 2,480 images, and the benign subset contains 5,429 images. These images are collected from 82 patients including four magnification factors, which are 40×, 100×, 200×, and 400×. The number of images for each magnification factor is shown in Table 1. Samples of these images with 400× amplification factor are shown in Fig. 1.

**Table 1:** The distribution of images among each magnification factor of BreakHis Dataset.

Magnification	Benign	Malignant	Total
**40×**	625	1,370	1,995
**100×**	644	1,437	2,081
**200×**	623	1,390	2,013
**400×**	588	1,232	1,820
**Total**	2,480	5,429	7,909
**#Patients**	24	58	82

#### ICIAR 2018 dataset

**Figure 2:**
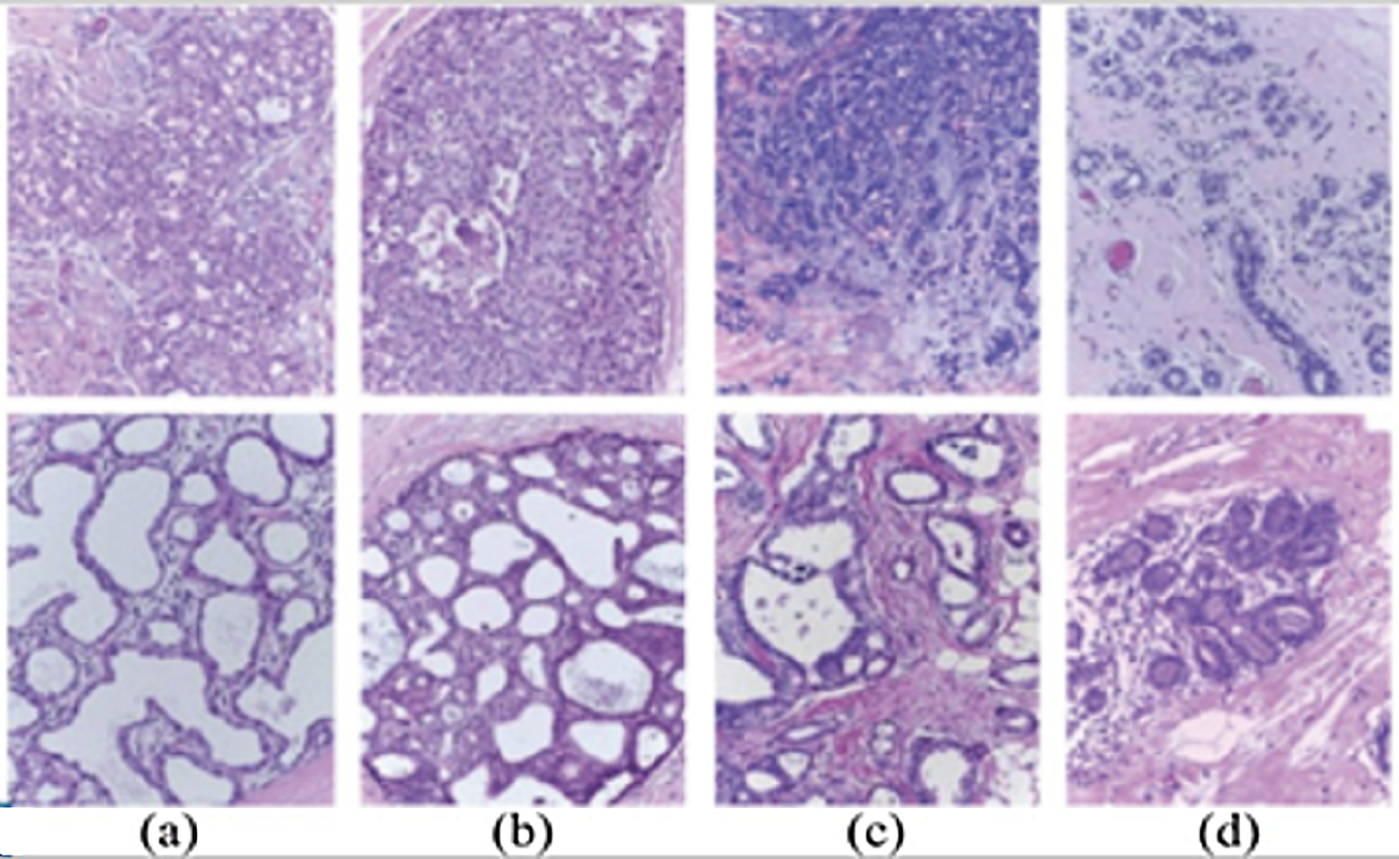
Examples of microscopic breast cancer histological images of ICIAR 2018 dataset: (A) benign, (B) in situ carcinoma, (C) invasive carcinoma, (D) normal.

The ICIAR 2018 dataset consists of a total of 400 Hematoxylin and eosin (HE) stained pictures of breast histology microscopic images having a resolution of 2,048 × 1,536 pixels. Both images are digitized under the same conditions of processing, with a magnification of 200× and pixel size of 0.42 m × 0.42 m. Each image is labeled with one of the four balanced classes: benign, in situ carcinoma, invasive carcinoma, and normal. Each class has 100 images as shown in Fig. 2. Two expert pathologists performed the image-wise annotation of ICIAR 2018 images.

### Convolutional neural networks

Convolutional neural networks (CNN) are DL techniques that are widely used with medical images to perform diagnosis (Jin et al., 2020; Ravì et al., 2016; Attallah, Ragab & Sharkas, 2020). We use five CNN architectures in this paper. Such networks include AlexNet, GoogleNet, ResNet-50, ShuffleNet, and Inception-ResNet V2 constructions. The number of layers and input/output size of the five networks used in the proposed method are shown in Table 2.

**Table 2:** The number of features extracted and the number of layers of the architecture of the five CNNs.

CNN Structure	Number of layers	Size of input
**GoogleNet**	22	224 × 224
**AlexNet**	8	227 × 227
**ResNet-50**	50	224 × 224
**ShuffleNet**	50	224 × 224
**Inception-ResNet V2**	164	229 × 229

#### ShuffleNet

Identifying significant features requires larger and deeper CNNs structures which need a huge amount of time to be trained (Girshick et al., 2014; Attallah, Ragab & Sharkas, 2020). To avoid this problem, a largely effective CNN called ShuffleNet was proposed by Zhang et al. (2018). ShuffleNet was initially produced to be used in fields that require small computation ability. The ShuffleNet construction consists of two new approaches called pointwise group convolution and channel shuffle. The pointwise group convolution employs convolution layers of size 1 × 1 kernel layer which reduce the time needed for training while attaining adequate performance. The shuffle operation supports the streaming of data along channels of features. This process allows a collection of convolution layers to manage the data of the input from different groupings (Attallah, Ragab & Sharkas, 2020).

#### AlexNet

AlexNet is a CNN architecture introduced in Krizhevsky, Sutskever & Hinton (2012). Its construction comprehends 23 layers corresponding to five convolutional layers, five ReLu layers, three pooling and two normalization layers, three FC layers, a softmax layer, and a classifying layer used to distinguish between images of 1,000 class labels (Attallah, Ragab & Sharkas, 2020; Attallah, Sharkas & Gadelkarim, 2020).

#### Inception-ResNet-V2

Inception-ResNet proposes residual links in the Inception architecture (Szegedy et al., 2016). It is a blend of the Inception block and the ResNet structure, where several convolutional filters of different sizes are pooled with residual connections. It was proposed to accelerate the training of the Inception networks inspired by the achievement of residual learning of ResNet. This mixture of the Inception block and ResNet blocks has significantly enhanced the performance of the architecture as well as the training speed. This network consists of 164 layers.

**Table 3:** The number of images after augmentation for each magnification level of BreakHis dataset.

Magnification factor	Benign	Malignant	Total
40×	18,750	41,100	59,850
100×	19,320	43,110	62,430
200×	18,690	41,700	60,390
400×	17,640	36,960	54,600
**Total**	74,400	162,870	237,270

**Table 4:** The number of images after augmentation for ICIAR 2018 dataset.

Magnification factor	Benign	Malignant	Total
400×	200	200	400
**Total**	6,000	6,000	12,000

#### GoogleNet

GoogleNet architecture was created in 2014. GoogleNet’s structure is much deeper than AlexNet. Its architecture depends on the Inception block. This block drops some parameters from a network. Thus, GoogleNet has offered a faster convergence than AlexNet (Attallah, Ragab & Sharkas, 2020). GoogleNet structure received first place in the ImageNet Large-Scale Visual Recognition Challenge (ILSVRC14) in 2014 (Attallah, Ragab & Sharkas, 2020; Szegedy et al., 2015). Finally, an FC layer is placed just before the output layer (Attallah, Ragab & Sharkas, 2020).

#### ResNet-50

ResNet architecture is commonly used in many areas due to its outstanding performance. Its core element is the residual element proposed in He et al. (2016). This element block proposes small links inside each layer of the convolution, the help ResNet to hop some layer of convolution (Attallah, Ragab & Sharkas, 2020). For this reason, ResNet architecture is believed to be more efficient than GoogleNet and AlexNet and (Talo et al., 2019; He et al., 2016; Ragab & Attallah, 2020; Attallah, Ragab & Sharkas, 2020). In this study, ResNet-50 is used which consists of 49 convolutional layers and one FC layer.

### Auto-encoder

The autoencoder (AE) is a class of unsupervised DL learning algorithms. An AE determines significant lower-dimensional representations from unlabeled data by mapping inputs and outputs (Wang, Yao & Zhao, 2016). It consists of two main parts: an encoder that maps the input data into a code. This process is made by an internal (hidden) layer that describes a code used to represent the input. The other part is a decoder that maps the code to reconstruct the original input. In other words, an AE acquires an illustration (encoding) for a set of data, normally for dimensionality reduction, by learning the network to neglect the redundant information that exists in the input data. Besides, the reduction side, a reconstructing side is trained, where the AE attempts to produce a demonstration as close as possible to its original data from the reduced encoded data.

### Proposed Histo-CADx system

**Figure 3:**
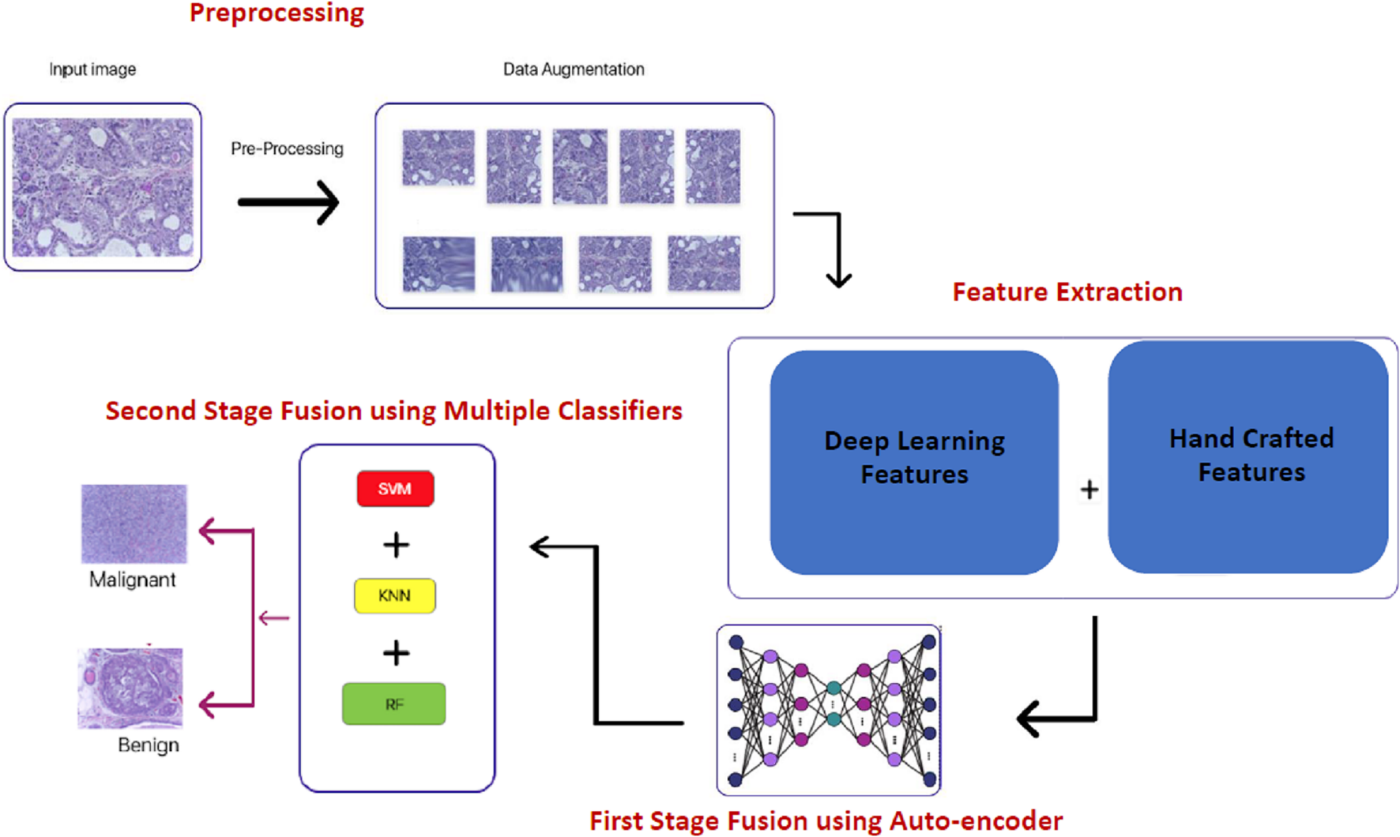
The block diagram of the proposed Histo-CADx.

A novel hybrid CADx system namely, Histo-CADx, is proposed to diagnose BC from histopathological images. Histo-CADx has four phases including image preprocessing, feature extraction, first stage fusion, and second stage fusion. In the image preprocessing step, augmentation is used to increase the size of the dataset and balance the number of images among classes. In the feature extraction, five different CNNs are used as feature extractors including (ResNet-50, AlexNet, GoogleNet, Inception-ResNet, and Shuffle-Net). Also, two handcrafted feature sets are extracted by Wavelet Packet Decomposition (WPD) and Histogram of oriented gradients (HOG). In the first stage of the fusion, deep features and handcrafted features are fused using the AE technique. AE is capable of reducing the huge dimension of feature space generated by different feature extraction techniques of the previous step. Besides, AE lowers the computational cost of diagnosis. In this step, several fused feature sets are generated using sequential forward and backward searching strategies. The best combination of fused feature sets that could enhance the performance of the CADx system is determined. In the second stage of the fusion, three machine learning classifiers are trained with the feature sets generated in the previous step to improve the performance of the proposed CADx. These classifiers include support vector machine (SVM), Random Forest (RF), and Quadratic discriminate analysis (QDA). The outcomes of these three classifiers are fused using a majority voting procedure. Figure 3 shows a block diagram of the proposed Histo-CADx.

#### Image preprocessing

In this step, images of both datasets are resized according to the size of the input layer shown in Table 2. Then, these images are augmented. Augmentation is a crucial procedure that is usually performed to increase the number of images in a dataset. This technique is carried out since the trained models probably suffer from over-fitting (Ravì et al., 2016). Over-fitting occurs when the classification parameters correspond too closely to the training data and may therefore fail in predicting new cases. Augmenting the images is done in this paper using random resizing, shearing, rotating, cropping, zooming, shifting, and flipping techniques. The number of images after the augmentation process for BreakHis dataset is shown in Table 3. The number of images after the augmentation process for ICIAR 2018 dataset is shown in Table 4.

#### Feature extraction

In this step, two paradigms are used for feature extraction. The first one uses traditional feature extraction approaches such as WPD and HOG. The second one employs spatial DL features pulled out from the ShuffleNet, AlexNet, GoogleNet, ResNet CNNs as suggested by Ragab & Attallah (2020), and Inception ResNet CNN. These feature extraction approaches are illustrated in the subsequent subsections.

##### Handcrafted feature extraction

Feature extraction techniques based on texture analysis are widely utilized in the medical field (Ragab & Attallah, 2020; Attallah, 2021). These approaches commonly rely on the textural features located in the medical image. The wavelet packet decomposition (WPD) and the histogram of oriented gradients (HOG) are examples of these approaches that usually achieve acceptable performance particularly if they are merged (Nailon, 2010; Lahmiri & Boukadoum, 2013). Thus, WPD and HOG approaches are utilized in this paper.

**Figure 4:**
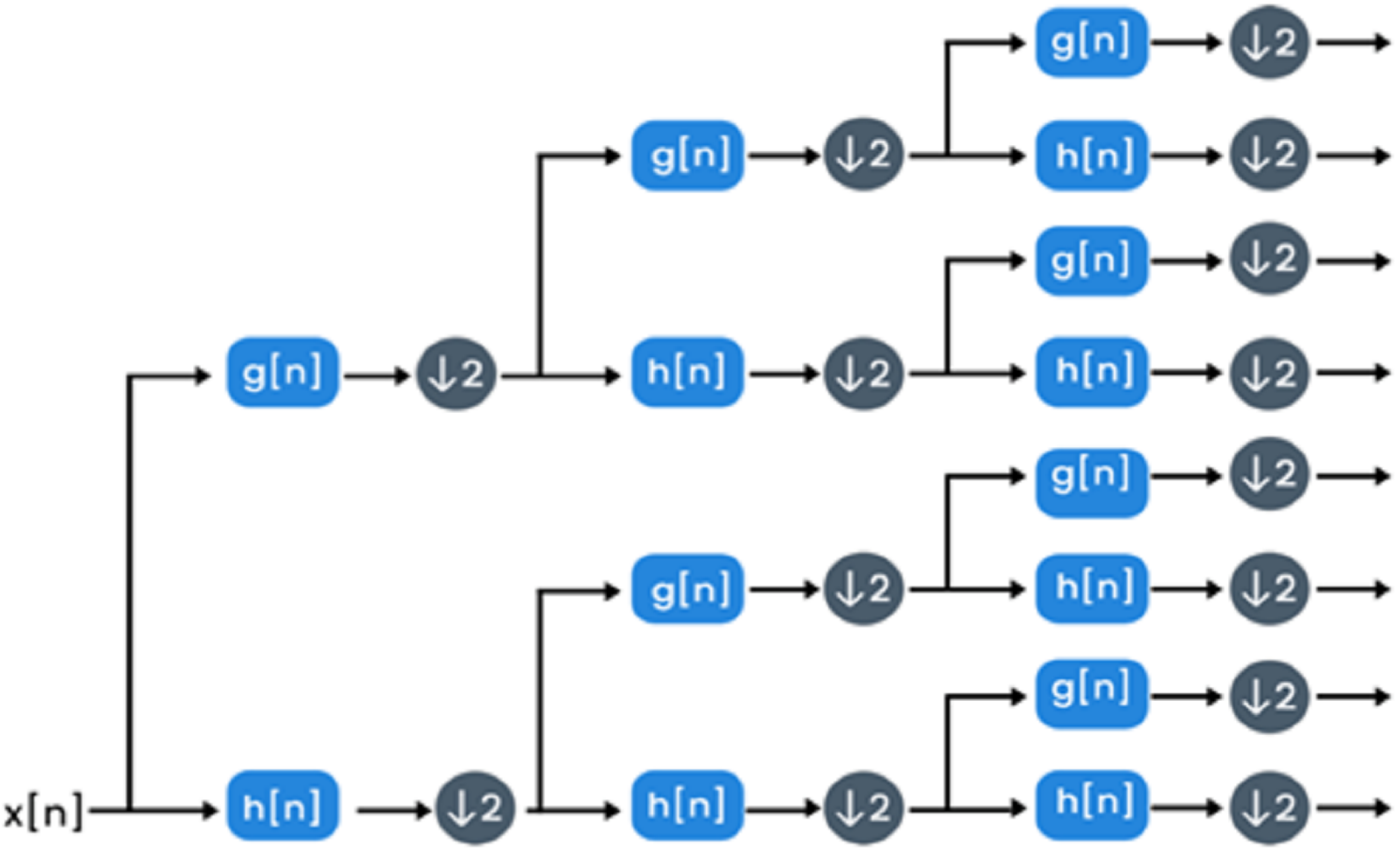
The decomposition procedure of WPD.

**WPD:** The decomposition process of the wavelet packet is the discrete wavelet transform extension. WPD is used to explore both images and signals. This extension can provide multi-level signal/image conversion from the time domain to the time-frequency domain. WPD process is made by passing an image/signal x(n) to a high and low pass filter denoted as g(n) and h(n) respectively (Attallah et al., 2020) and then down-sampling by 2 is done. The filtering process is equivalent to convolving low pass and high pass filters with a signal/image. Two sets of coefficients will be produced after this step called details and approximation coefficients. Afterward, both the approximation and details coefficients are further analyzed by entering low and high pass filters once again followed by down-sampling by 2. Once more, the details and approximation coefficients may be analyzed further and enter low pass and high pass filters. The procedure of filtering followed by down-sampling is called a decomposition level. Figure 4 shows the decomposition procedure of WPD. In Histo-CADx, 4-WPD decomposition stages are applied to the images of the two datasets. A Shannon basis function is utilized. The 4^th^ decomposition level usually improves the performance of classification (Ragab & Attallah, 2020). All the coefficients of the 4^th^ level were taken as features. The size of features extracted after WPD is 1,067.

**HOG:** a well-known feature extraction technique that relies on calculating the gradient alignments located in several regions of the region of interest (ROI) (Newell & Griffin, 2011; Anwar et al., 2020). This process is done through 5 stages which are:

1. Global image normalization.
2. Gradient measurement in x and y directions.
3. Computing histograms of gradients.
4. Standardization across blocks.
5. Flapping in a vector feature.

The first stage incorporates a normalization of a global image designed to reduce the effects of local variations in shadowing and lighting. The second stage calculates the gradients of the first-order image. This stage captures contour, outline, and some details about texture while providing additional resistance to variations in illumination. The third stage attempts to create an encoding scheme that is sensitive to the content of the local image. The frame of an image called “cells” is divided into small areas of space. A local 1-D gradient or edge orientation histogram is collected for each cell. The orientation histogram divides the gradient angle into a set number of predetermined bins. The cell’s pixel gradient magnitudes are used to determine the orientation histogram. The fourth stage standardizes local cell groups and normalizes their total responses before progressing to the next stage. These responses are calculated by accumulating a measure of local “energy” histogram over local cell groups that we call “blocks.” The result is used to standardize every block cell. The final step collects the HOG descriptors in a combined vector. The size of feature space using this type of feature extraction is equal to 2,156.

##### Deep learning feature extraction

The CNNs constructed can perform feature extraction or classification. This paper extracts spatial features from the third maximum pooling layer, fourth pooling layer, “global average pooling 2D layer”, and average pooling layers of the AlexNet, GoogleNet, ResNet-50, ShuffleNet, and Inception-ResNet CNNs. The size of the feature space extracted via these CNNs is equal to 7,852, 5,000, 6,034, 3,520, and 4,600 for Inception- ResNet, ResNet-50, ShuffleNet, AlexNet, and GoogleNet respectively

#### First fusion stage

This stage includes feature fusion. It fuses the DL features along with handcrafted features. This stage is split into three phases namely, feature set generation, feature set search, and feature set fusion.

- **Feature Sets Generation Phase**

In this phase, the DL and handcrafted features extracted in the previous step are used individually as an input to the classifier. The classification accuracy of the classifier is used to rank each feature set in descending order according to the accuracy achieved by each feature set individually. This rank is used to generate several feature sets. The feature sets are examined and selected in the next phases.

- **Feature Set Selection Phase**

In this phase, the feature sets generated in the previous phase are searched to select the finest mixture of features that has the largest impact on the accuracy of BC diagnosis. This search procedure is done using sequential forward and backward searching strategies. In the forward strategy, the search starts with the first feature set which has the highest accuracy in the previous phase. Then, each subsequent feature set is added iteratively, if the feature set added improved the performance of the CADx, then it is kept, else it is neglected. This process is repeated until all feature sets are examined. Conversely, in the backward strategy, the search starts including all features and then removing the feature set with the lowest rank (smallest accuracy in the previous phase). Afterward, every preceding feature set (having a higher rank) is iteratively removed, if the eliminated feature set enhances the performance of the CADx, otherwise, it is not removed.

The features generated for the BreakHis dataset in case of sequential forward strategy are denoted as FS-1-BH-FW to FS-7-BH-FW; where FS is feature set, BH stands for BreakHis dataset, FW stands for forward, and 1 to 7 represents the number of the feature set generated. FS-1-BH-FW corresponds to the first feature sets containing only one feature which have the highest rank. On the other hand, the features generated for the BreakHis dataset in the case of sequential backward strategy are denoted as FS-1-BH-BW to FS-7-BH-BW, where BW refers to backward search. FS-1-BH-BW feature set represents the fusion of all features (7 feature sets).

Similarly, for ICIAR 2018 dataset, the feature sets produced in the forward strategy are FS-1-ICIAR-FW to FS-7-CIAR_FW where; FS stands for the feature set, ICIAR refers to the ICIAR 2018 dataset, FW stands for forward, and 1 to 7 represents the number of the feature set produced. FS-1-ICIAR-FW represents the first feature set containing only one feature type that has the highest rank. Whereas in the case of the backward strategy, the sets produced are FS-1-ICIAR-BW to FS-7-CIAR_BW, where BW stands for backward search. Note that, FS-1-ICIAR-BW is equivalent to the fusion of all the 7 features (5 extracted from the different CNNs + 2 handcrafted features)

- **Feature Sets Fusion Phase**

In this phase, feature sets that are examined using forward and backward strategies in the previous phase are fused using the AE DL technique. This phase is important to not only investigate the influence of fusing using the AE method but also to reduce the huge dimension resulting from the fusion of the feature sets.

#### Second fusion stage

This stage is a classifier-based fusion, where a multiple classifier system (MCS) is constructed. The MCS increases the power of each classifier and generally tends to beat the performance of individual classifiers in the pool of classifiers. The MCS is able to evade the likelihood of weak classification outputs produced from a certain inaccurate model (Attallah et al., 2017b). In this stage, three machine learning classifiers including SVM, RF, and QDA are trained using the feature sets generated in the previous step after being fused and reduced by the AE. The outputs of these classifiers are combined using majority voting.

## Experimental Set Up

### Measures of assessment

The capacity of Histo-CADx f is determined using measures including the F1-score, accuracy, precision, specificity, and sensitivity. The formulas used to calculate such metrics are shown in the equations below (1)–(5).

(1) $$Accuracy=\frac{TP+TN}{TN+FP+FN+TP}$$

(2) $$Sensitivity=\frac{TP}{TP+FN}$$

(3) $$Specificity=\frac{TN}{TN+FP}$$

(4) $$Precision=\frac{TP}{TP+FP}$$

(5) $$F1-Score=\frac{2\times TP}{\left(2\times TP\right)+FP+FN}$$

TP is the sum of accurately classified benign images, TN is the sum of correctly classified malignant images. FP is the total summation of malignant cases that were not classified properly. FN is the total summation of benign cases that were not classified properly.

### Parameters setting

In the proposed system, we used 70% of the data for training and 30% of the data for testing. To improve the generalization of classification when testing new cases, we used non-overlapping sets of images for training and testing. First, the data was split into training and testing according to persons, not randomly. To avoid overfitting, we made sure that the images of persons chosen in the training set are only included in the training set, and not included in the testing set. While the images that belong to persons chosen in the testing set are only included in the testing set. Afterward, the augmentation process was accomplished on these disjoint training and testing sets.

Parameters setting is an initial and important step that affects the performance of the CNNs in general. An epoch is defined as the complete one pass through the full training dataset. The learning rate is a parameter that determines how much an updating step influences the current value of the weights. While weight decay is an additional term in the weight update rule that causes the weights to exponentially decay to zero if no other update is scheduled. In the current model, the epochs’ number is set to be 20. The initial learning rate is set to be 0.045. The weight decay and momentum were both set to 0.9. Default values were used for the remaining parameters. Finally, stochastic gradient descent with momentum (SGDM) was used for optimization.

## Results

In this study, we propose a novel CADx system based on two cascaded fusion stages. The first fusion stage is a feature fusion process that investigates the influence of fusing multiple CNNs and two handcrafted features in classifying BC. This stage also searches for the best combination of features that enhances the classification results. On the other hand, the second stage is a classifiers-based fusion procedure, where a MCS is created and the outputs of three classifiers are fused using majority voting. The classification results of Histo-CADx are discussed in this section.

### First stage fusion results of Histo-CADx

During the first fusion stage, five spatial features are mined from several CNNS along with two handcrafted features. Three experiments are carried out to verify that feature fusion can enhance the results of the proposed CADx. In experiment (1), the features extracted by different methods are used to train separate SVM, QDA, and RF classifiers. Experiment (2) represents the fusion of all features including the DL and handcrafted features using the AE DL method. This experiment is done to test if feature fusion with AE could improve the accuracy of the CADx constructed with the three distinct classifiers. In experiment (3) the impact of using sequential forward and backward searching strategies for feature fusion based on AE is examined. The results of these three experiments are shown in this subsection.

#### Experiment (1) results

The results of experiment (1) are shown in Tables 5 and 6 for the BreakHis and ICIAR datasets respectively. Table 5 shows that in the case of the 40× magnification factor for the BreakHis dataset, the highest accuracy of 96.31% is achieved using the SVM classifier trained with the features extracted from the Inception-ResNet V2 CNN. Whereas, for the 100× magnification, the RF classifier trained with the DL features of the Inception-ResNet V2 CNN attains the maximum accuracy of 96.83%. Similarly, for the 200× magnification factor, the RF classifier constructed with the DL features of the Inception-ResNet V2 CNN reaches the peak accuracy of 94.94%. While, for the 400× magnification factor, the greatest accuracy of 92.994% is obtained using the SVM classifier. These results indicate that the highest accuracy among the four magnification factors is achieved using the 200× with RF classifier built with the Inception-ResNet V2 features.

**Table 5:** The classification accuracy of each individual feature using SVM, RF, and QDA classifiers for BreakHis dataset.

Features	SVM	RF	QDA
**Magnification Factor 40×**			
**Inception-Resnet V2**	96.31	95.72	93.04
**ResNet-50**	95.65	91.33	93.78
**GoogleNet**	94.29	95.09	92.58
**AlexNet**	91.98	92.44	94.84
**ShuffleNet**	86.79	88.02	90.05
**HOG**	90.75	93.01	89.99
**WPD**	88.91	92.27	91.77
**Magnification Factor 100×**			
**Inception-Resnet V2**	95.33	96.83	93.09
**ResNet-50**	93.11	94.71	95.35
**GoogleNet**	92.99	94.89	90.84
**AlexNet**	88.69	91.35	92.65
**ShuffleNet**	86.25	88.93	87.82
**HOG**	91.24	90.49	80.09
**WPD**	88.77	90.76	86.31
**Magnification Factor 200×**			
**Inception-Resnet V2**	92.22	94.94	90.52
**ResNet-50**	90.43	92.60	93.00
**GoogleNet**	90.45	92.20	89.11
**AlexNet**	88.51	90.94	87.31
**ShuffleNet**	85.13	81.46	78.99
**HOG**	87.46	85.31	88.45
**WPD**	82.21	87.32	79.45
**Magnification Factor 400×**			
**Inception-Resnet V2**	92.994	90.122	87.432
**ResNet-50**	89.456	88.321	90.322
**GoogleNet**	86.666	89.300	87.199
**AlexNet**	84.486	87.432	88.444
**ShuffleNet**	82.345	81.111	80.340
**HOG**	84.776	85.241	83.888
**WPD**	85.828	83.102	81.442

**Table 6:** Classification accuracy (%) for individual features using SVM, RF, and QDA classifiers for ICIAR 2018 dataset.

Features	SVM	RF	QDA
**Inception-Resnet V2**	83.984	81.746	79.833
**ResNet -50**	76.537	79.345	78.155
**GoogleNet**	88.935	89.232	86.831
**AlexNet**	75.927	77.124	79.485
**ShuffleNet**	80.324	82.542	85.534
**HOG**	81.143	83.721	84.443
**WPD**	76.639	77.993	74.235

Table 6 demonstrates the classification accuracies of the SVM, RF, and QDA classifiers trained individually with features extracted from the five CNNs for the ICIAR 2018 dataset. It is obvious from the table that the peak accuracy of 89.232% is attained using the RF classifier built with GoogleNet features followed by 88.935% and 86.831% achieved using the SVM and QDA classifiers respectively constructed with GoogleNet features as well.

#### Experiment (2) results

The results of experiment (2) are shown in Tables 7 and 8 for the BreakHis and ICIAR datasets respectively. Table 6 displays the BreakHis dataset’s classification accuracy for all fused features using the SVM, RF, and QDA classifiers. The table indicates that fusing all features has improved the accuracy attained using SVM, RF, and QDA classifiers for all magnification factors compared to individual features of the Inception-ResNet except for the SVM classifier at the 400× magnification factor. The highest accuracy (97.848%) amongst the four magnification factors is achieved using the RF classifier trained with all fused features of the 40×.

**Table 7:** Classification accuracy (%) for all fused features using SVM, RF, and QDA classifiers for the BreakHis dataset.

Features	SVM	RF	QDA
**Magnification Factor 40×**			
**Inception-ResNet-50**	96.31	95.72	93.04
**All fused features**	96.499	97.848	94.65
**Magnification Factor 100×**			
**Inception-ResNet-V2**	95.33	96.83	93.09
**All fused features**	96.63	97.63	94.65
**Magnification Factor 200×**			
**Inception-ResNet-V2**	92.994	90.122	87.432
**All fused features**	95.71	92.84	90.39
**Magnification Factor 400×**			
**Inception-ResNet-V2**	92.994	90.122	87.432
**All fused features**	89.436	92.543	87.902

**Table 8:** Classification accuracy (%) for all fused features using SVM, RF, and QDA classifiers for the ICIAR 2018 dataset.

Features	SVM	RF	QDA
**GoogleNet**	88.935	89.232	86.831
**All fused features**	92.034	94.309	93.324

In Table 8, we show that SVM, RF, and QDA classifiers produce accuracies of 92.034%, 94.309%, and 93.324% after fusing all DL and handcrafted features of the ICIAR 2018 dataset. These accuracies are compared with the individual GoogleNet feature set which accomplished the highest accuracy in experiment 1. We can conclude that merging all DL and texture-based features have enhanced the performance of the SVM, RF, and QDA classifiers for the ICIAR 2018 dataset.

#### Experiment (3) results

**Figure 5:**
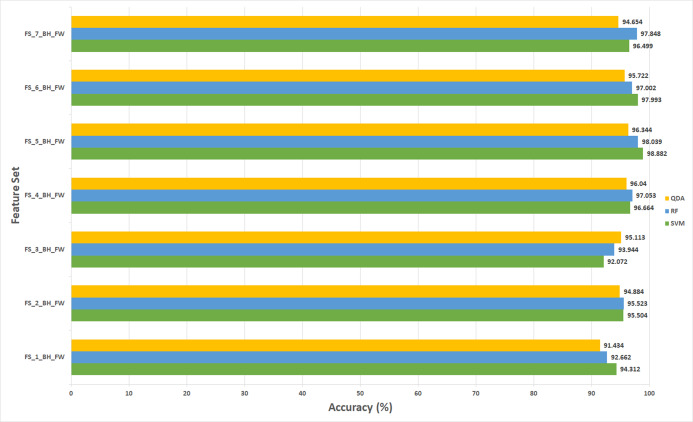
The classification accuracies of the first stage of Histo-CADx for the 40× magnification factor of the BreakHis dataset using the sequential forward searching strategy.

**Figure 6:**
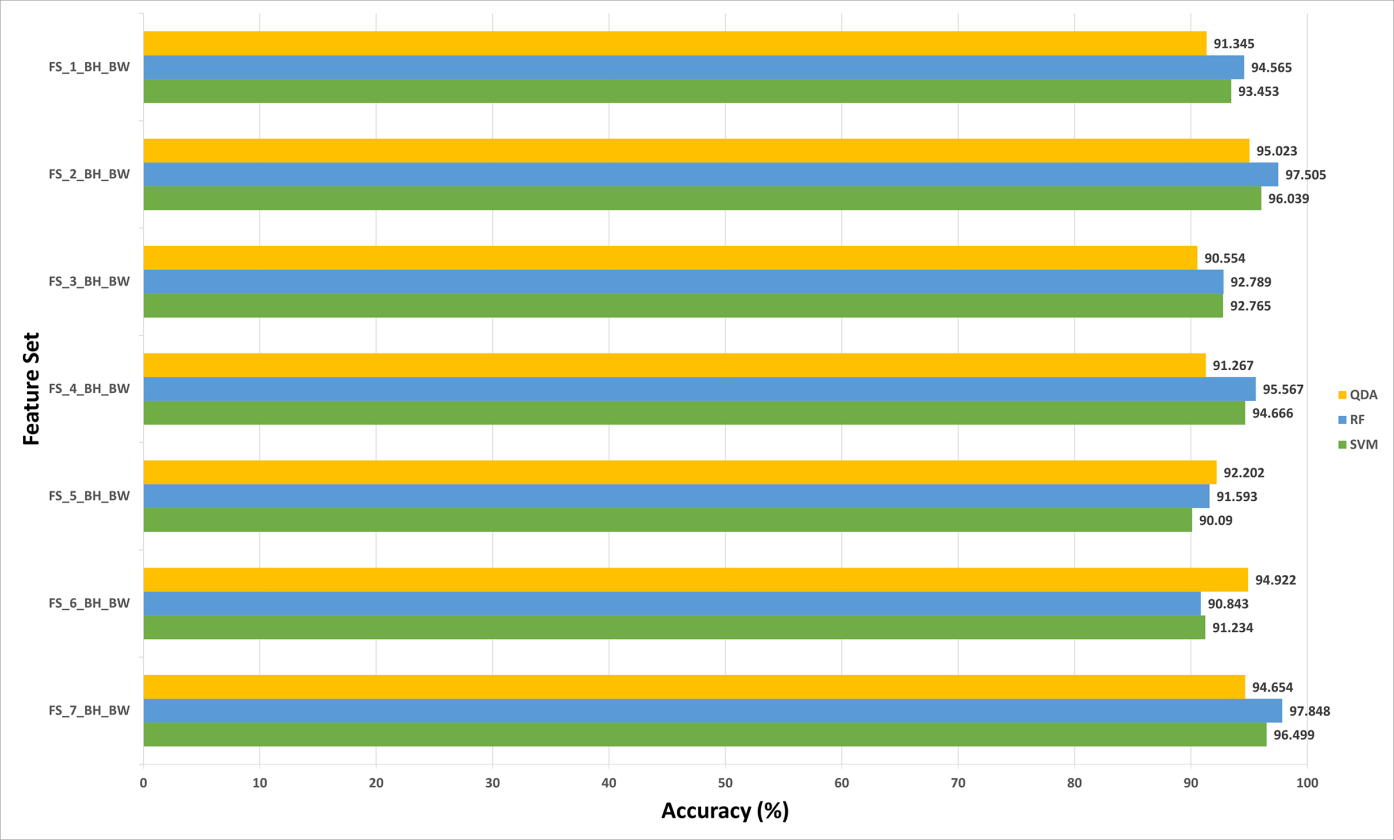
The classification accuracies of the first stage of Histo-CADx for the 40× magnification factor of the BreakHis dataset using the sequential backward searching strategy.

**Figure 7:**
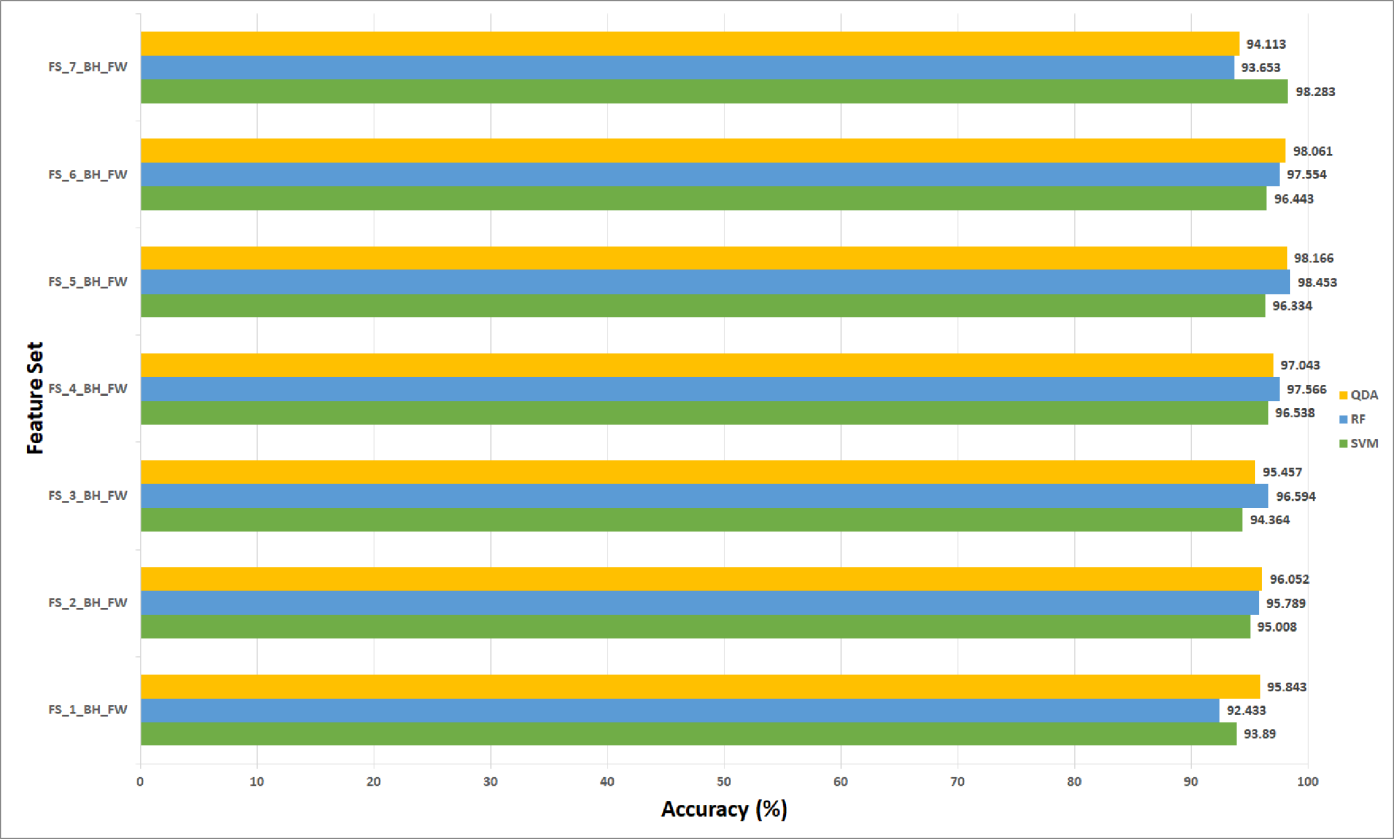
The classification accuracies (%) of the first stage of Histo-CADx for the 100× magnification factor of the BreakHis dataset using the sequential forward searching strategy.

**Figure 8:**
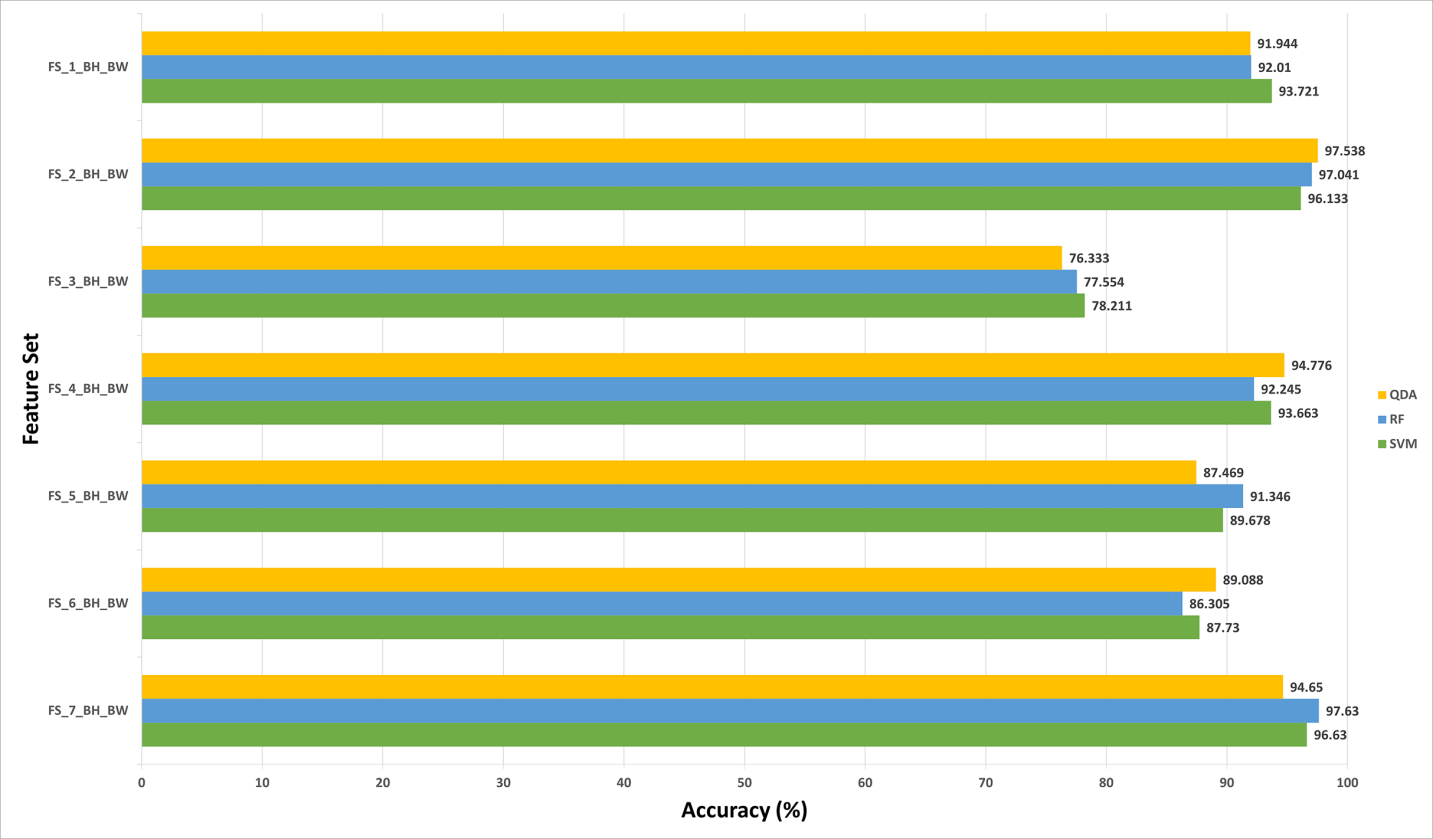
The classification accuracies (%) of the first stage of Histo-CADx for the 100× magnification factor of the BreakHis dataset using the sequential backward searching strategy.

**Figure 9:**
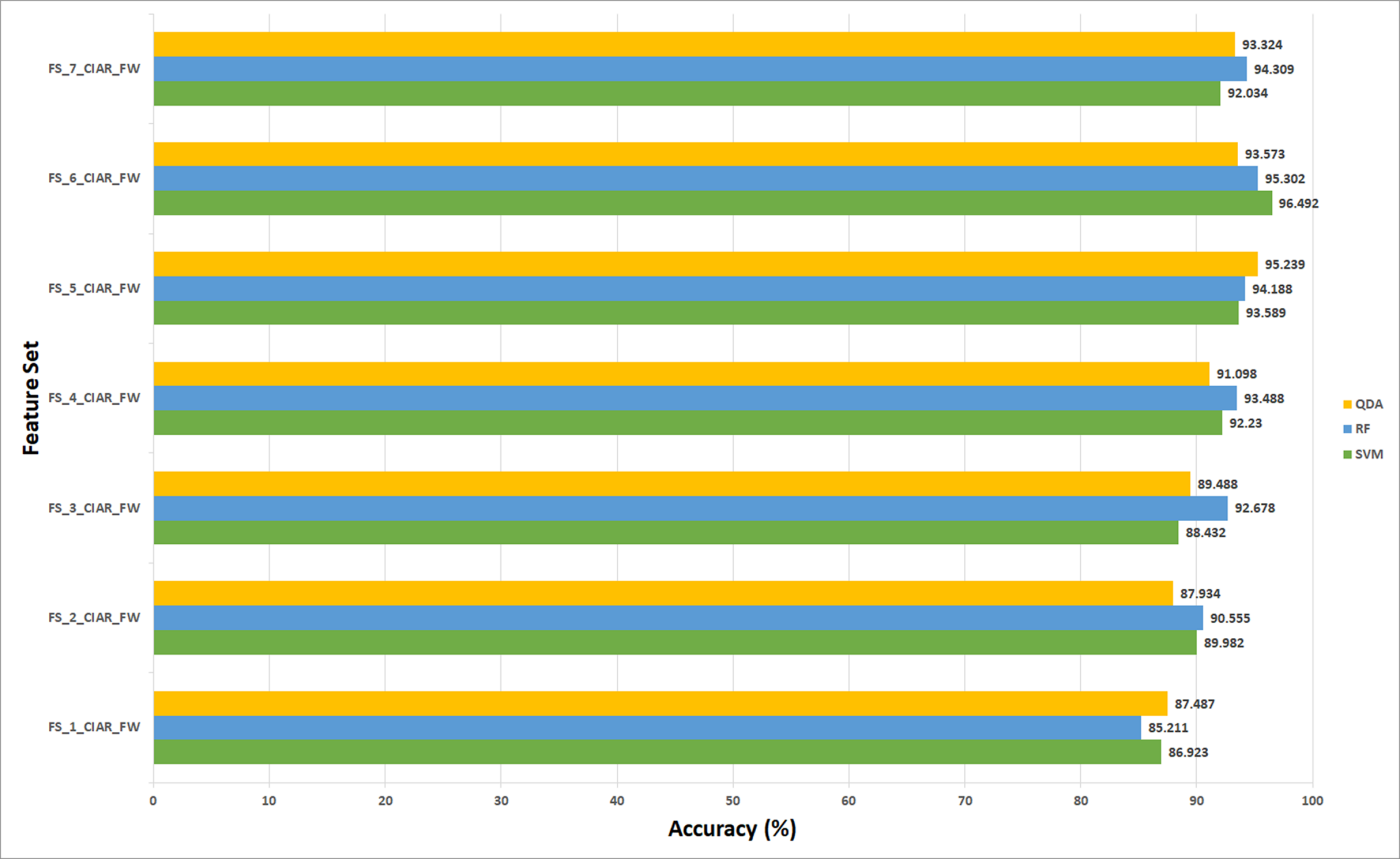
The classification accuracies (%) of the first stage of Histo-CADx for the ICIAR 2018 dataset using the sequential forward-searching strategy.

**Figure 10:**
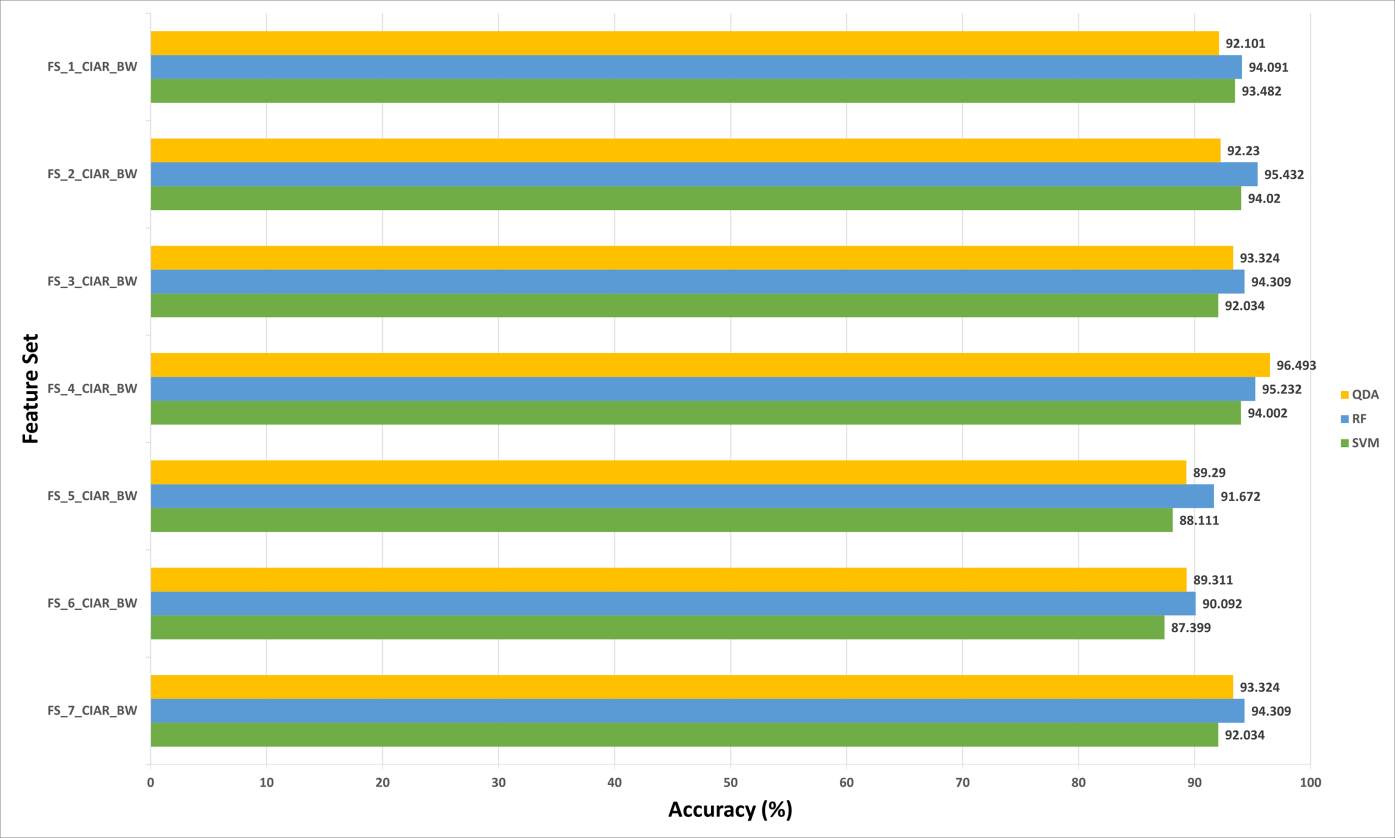
The classification accuracies (%) of the first stage of Histo-CADx for the ICIAR 2018 dataset using the sequential backward searching strategy.

The results of experiment (3) are shown in Figs. 5 to 8 for 40× and 100× magnification factors of the BreakHis dataset and Fig. 9 and 10 for the ICIAR dataset. The results of the 40× and 100× magnification factors of the BreakHis dataset are displayed here as they achieved the highest performance in the previous experiments. Figure 5 shows that the peak accuracy of 98.882% is achieved using the SVM classifier trained with the feature set FS-5-BH-FW (using forward strategy) representing the fused features of the Inception-ResNet V2, ResNet-50, GoogleNet, AlexNet, and HOG extracted from the 40x magnification factor of the BreakHis dataset. On the other hand, the subset which attained the peak accuracy for the backward search strategy of the BreakHis dataset is FS-7-BH-BW. This subset attains an accuracy of 97.848% as shown in Fig. 6 using the RF classifier constructed with the fused features of the Inception-Resnet V2, ResNet-50, GoogleNet, AlexNet, ShuffeNet, WPD and HOG pulled out from the 40× magnification factor of the BreakHis dataset.

For the 100× magnification factor of the BreakHis dataset, the feature subset selected using the forward strategy is FS-5-BH-FW achieving an accuracy of 98.453% as shown in Fig. 7. This result is obtained using the RF classifier. Whereas, for the backward strategy, the chosen feature set is FS-7-BH-BW attaining an accuracy of 97.63% as shown in Fig. 8. This feature set corresponds to fusing the features of the Inception-Resnet V2, ResNet-50, GoogleNet, AlexNet, ShuffeNet, WPD, and HOG.

Regarding the ICIAR 2018 dataset, the feature set elected during the forward strategy of the first stage of fusion is FS-6-ICIAR-FW. The number of fused features in FS-6-ICIAR-FW is 6 representing the fused features of GoogleNet +HOG + ShuffleNet + Inception-ResNet + AlexNet + WPD. This set result in an accuracy of 96.492% using the SVM classifier. Conversely, for the backward search FS-4-ICIAR-BW yielded the highest accuracy of 96.493% using the RF classifier. The fused features in FS-4-ICIAR-BW include GoogleNet, HOG, ShuffleNet, Inception-Resnet, and ResNet-50 features.

### Second stage fusion results of Histo-CADx

**Figure 11:**
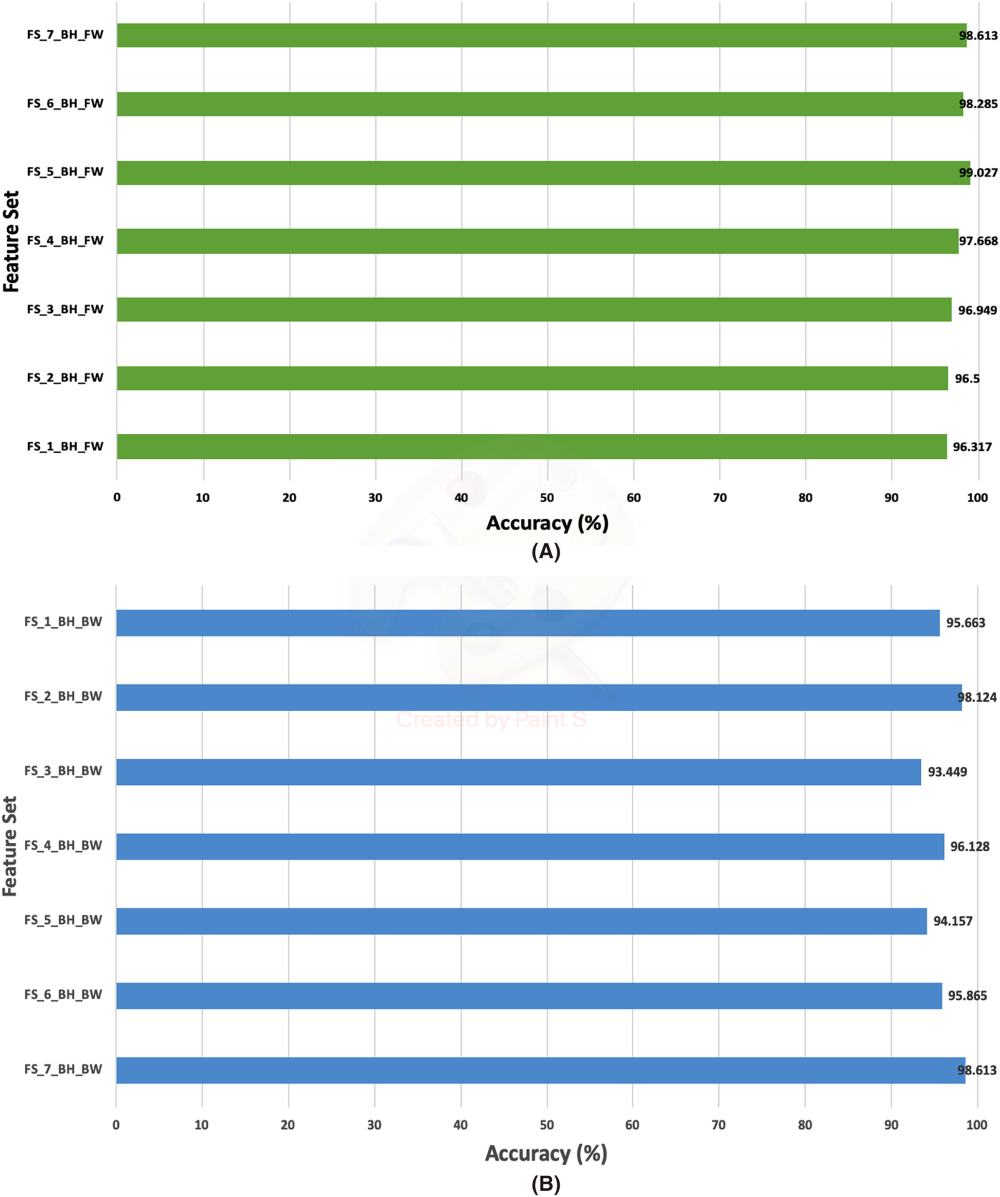
The classification accuracies of the second stage of fusion (MCS) of Histo-CADx for the 40× magnification factor of the BreakHis dataset using (A) the sequential forward searching strategy, (B) the backward searching strategy.

**Figure 12:**
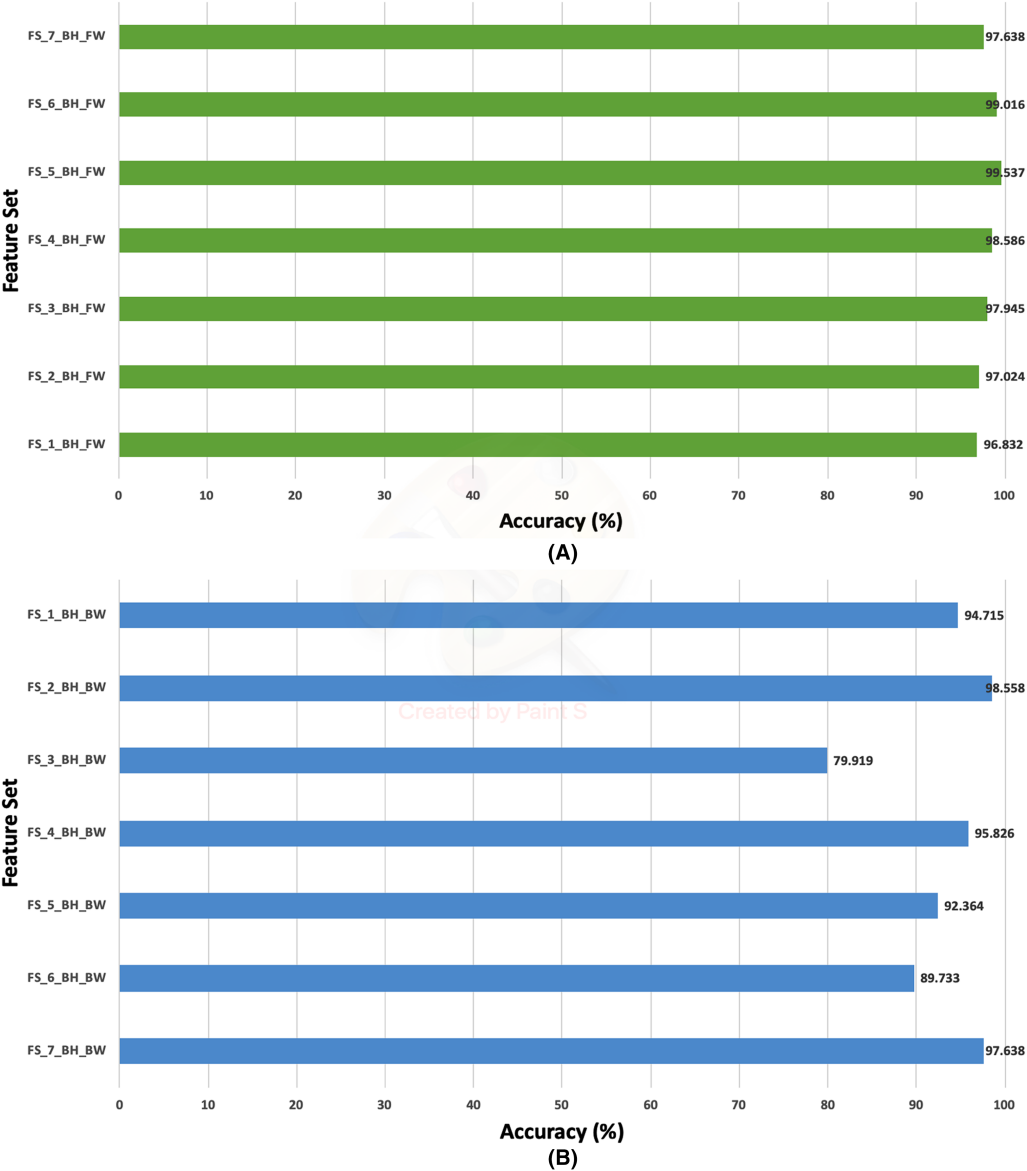
The classification accuracies of the second stage of fusion (MCS) of Histo-CADx for the 100× magnification factor of the BreakHis dataset using (A) the sequential forward searching strategy, (B) the backward searching strategy.

**Figure 13:**
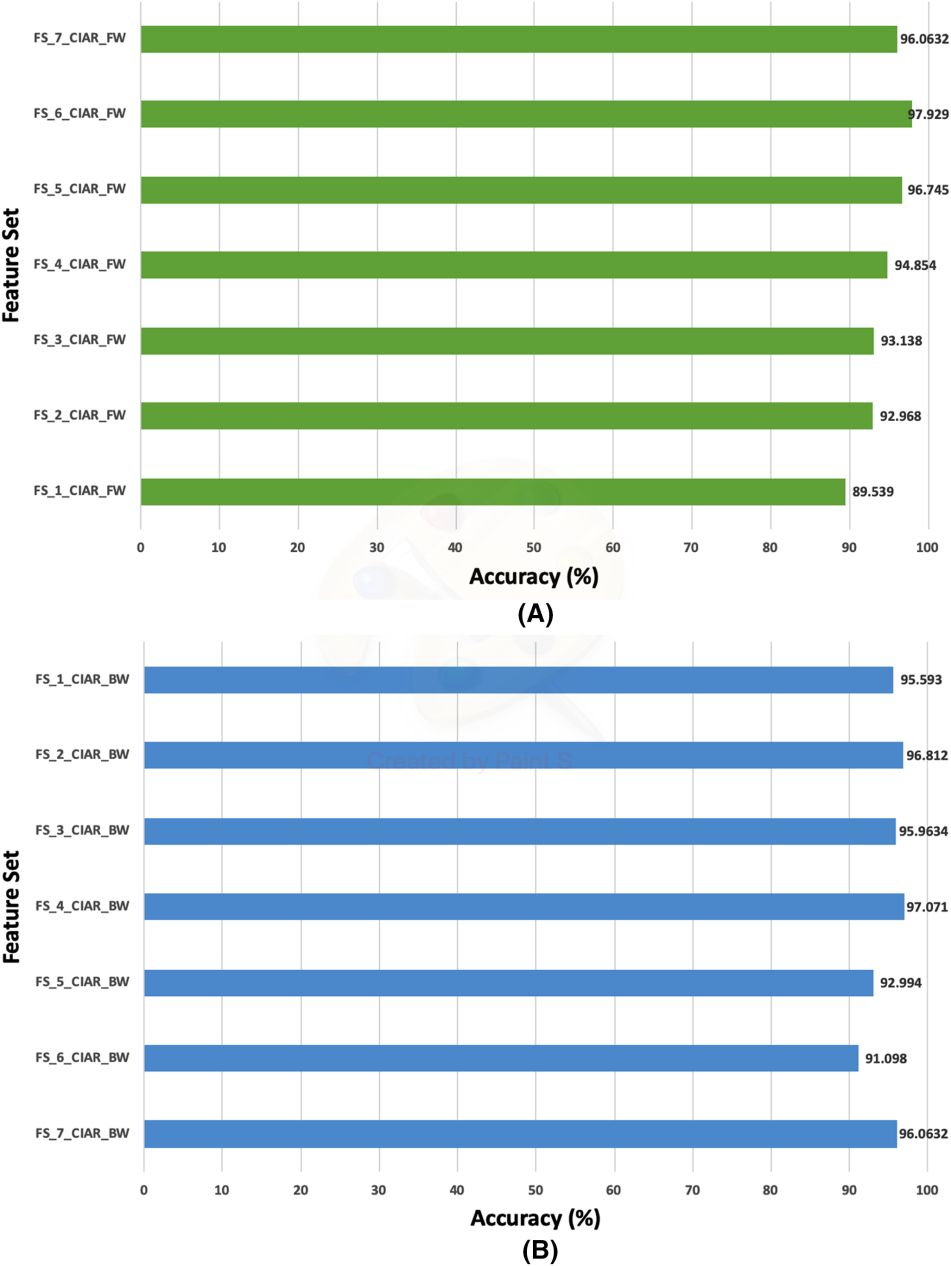
The classification accuracies of the second stage of fusion (MCS) of Histo-CADx for the ICIAR 2018 dataset using (A) the sequential forward searching strategy, (B) the backward searching strategy.

**Figure 14:**
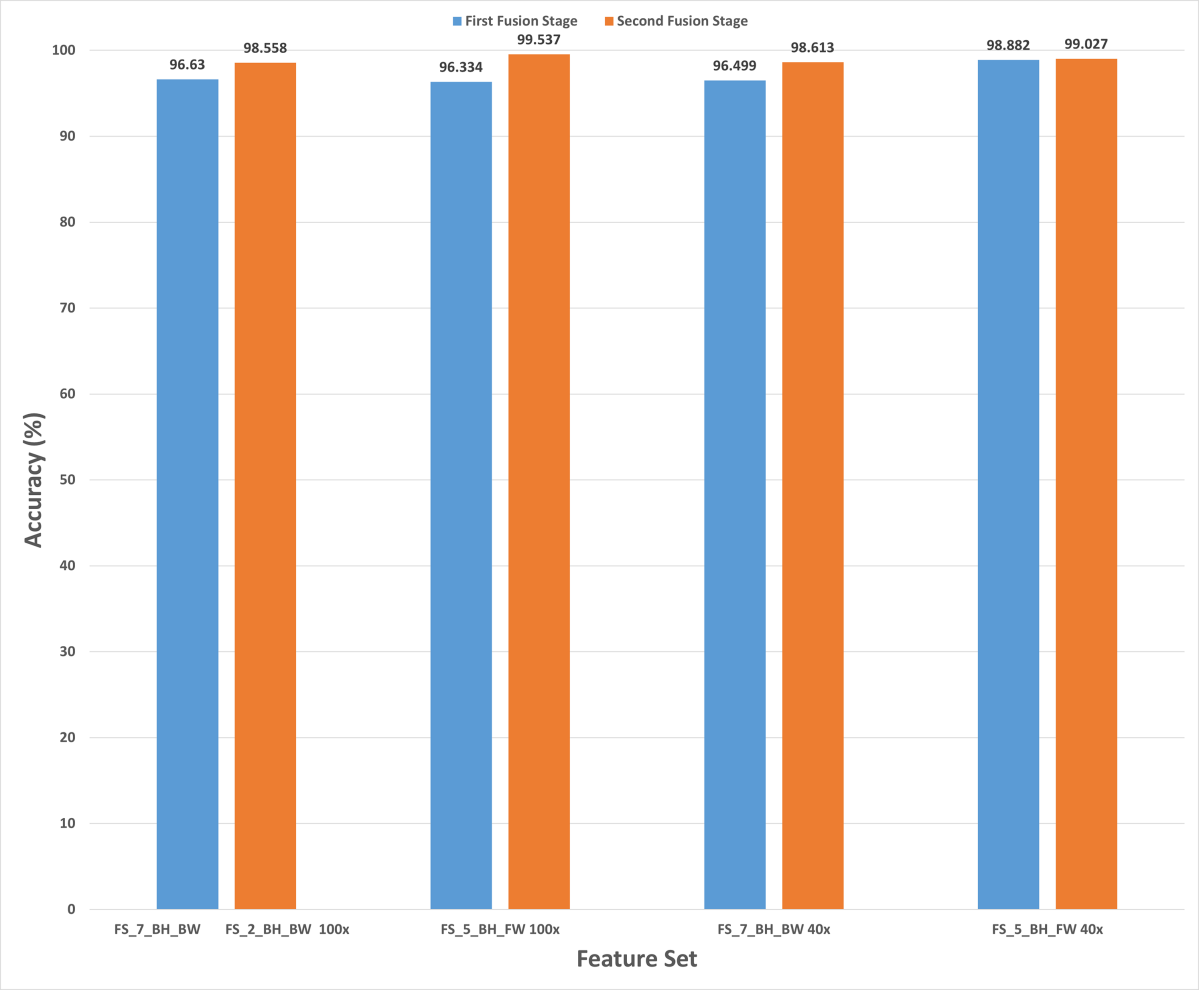
A comparison between the accuracies achieved using the first and second fusion stages of Hist-CADx constructed with the 40× and 100× magnification factors of the BreakHis dataset.

This section discusses the results of the second fusion stage for both datasets. It can be concluded that in the first fusion stage of Histo-CAD (experiment 3), the highest results were obtained using the 40× and 100× magnification factors. Therefore, these two magnification factors are further examined, and their results are shown here through Figs. 11 and 12. Also, the performance achieved using the ICIAR 2018 is displayed and discussed in Figs. 13 and 14. For the 40× magnification factor of the BreakHis dataset, the results in Fig. 11A indicate that in the case of the forward search, the highest accuracy of 99.027% is attained using FS_5_BH_FW. This set compromises the features of Inception-Resnet V2, ResNet-50, GoogleNet, AlexNet, and HOG. While, for the 100x, FS-7-BH-BW is chosen in the backward search as it corresponds to an accuracy of 98.613% as shown in Fig. 11B. This feature set contains the features of the Inception-Resnet V2, ResNet-50, GoogleNet, AlexNet, ShuffeNet, WPD, and HOG.

On the other hand, for the 100× of the BreakHis dataset, the forward search strategy ended up by choosing FS-5-BH-FW achieving an accuracy of 99.537% as indicated in Fig. 12A, whereas, in the backward search FS-2-BH-BW is selected, which yielded an accuracy of 98.558% as shown in Fig. 12B using the features of the Inception-Resnet V2, ResNet-50, GoogleNet, AlexNet, HOG, and WPD.

In the case of the ICIAR 2018 dataset, the forward search method resulted in electing FS_6_ICIAR_FW which obtained an accuracy of 97.929% using the six feature sets of the GoogleNet, HOG, ShuffleNet, Inception-ResNet, AlexNet, WPD as described in Fig. 13A. Whereas, for backward search, it is observed from Fig. 13B that the peak accuracy of 97.071% is attained using FS-4-ICIAR-BW. This feature set represents the GoogleNet, HOG, ShuffleNet, Inception-Resnet,and ResNet-50 features.

**Figure 15:**
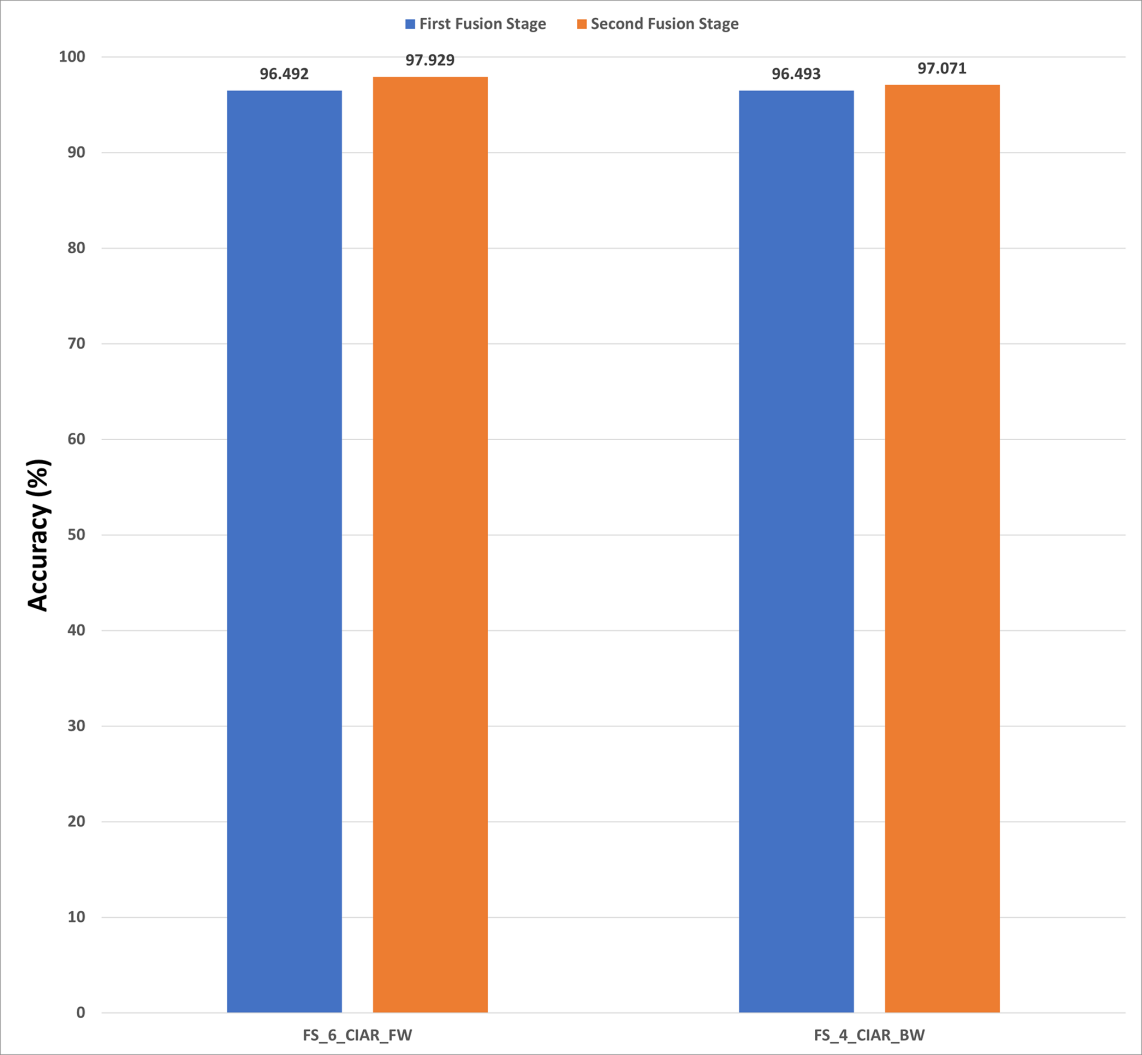
A comparison between the accuracies achieved using the first and second fusion stages of Histo-CADx constructed with the ICIAR 2018 dataset.

The results of the second fusion stage are compared with those of experiment 3 to verify that the second stage fusion has increased the performance of Histo-CADx. This comparison is shown in Figs. 14 and 15 for the BreakHis and ICIAR datasets respectively. Figure 14 proves that the forward strategy of the second stage fusion has improved the performance of Histo-CADx for the 40× and 100× magnification factors of BreakHis data. This is obvious as for the 40× and 100× of the BreakHis dataset, the accuracies of the FS-5-BH-FW selected feature set using forward strategy of the second stage fusion are 99.027% and 99.54% which are higher than that of 98.88% and 96.334% of the first fusion stage using the same feature set (FS-5-BH-FW). Moreover, the accuracies attained using the backward strategy of the second stage fusion of the feature sets FS-7-BH-BW and FS-2-BH-BW are 98.613% and 98.558% for the 40× and 100× magnification factors which are higher than that of 96.499% and 96.63% achieved in the first fusion stage using the feature set (FS-7-BH-BW).

Table 9 shows the performance metrics of the MCS of the second fusion stage created with the two feature sets selected using forward and backward strategies. The table specifies that the feature set FS-5-BH-FW selected using the forward strategy attained sensitivities of 0.99 and 0.9949, specificities of 0.9906 and 0.9959, precisions of 0.991and 0.9959, and F1-scores of 0.9905 and 0.9954 for the 40× and 100× magnification factors of the BreakHis dataset. Whereas the feature set FS-7-BH-BW and FS-2-BH-BW chosen using the backward strategy obtained sensitivities of 0.9879 and 0.9798, specificities of 0.9834 and 0.9656, precisions of 0.9889 and 0.9891, and F1-scores of 0.9884 and 0.9884 for the 40× and 100× magnification factors of the BreakHis dataset.

**Table 9:** Performance metrics for sequential forward and backward search stratigies of the second fusion stage using MCS classifiers for the BreakHis dataset.

Classifier	Sensitivity	Specificity	Precision	F1-Score
**Forward strategy (FS-5-BH-FW)**				
**MCS-40x**	0.99	0.9906	0.991	0.9905
**MCS-100x**	0.9949	0.9959	0.9959	0.9954
**Backward strategy (FS-1-BH-BW)**				
**MCS-40x**	0.9879	0.9834	0.9889	0.9884
**MCS-100x**	0.9798	0.9656	0.9891	0.9844

For ICIAR 2018 dataset, the results shown in Fig. 15 verify the second fusion stage has successfully improved the performance of Histo-CADx. For forward strategy, the accuracy attained using FS-6-ICIAR-FW has been enhanced from 96.492% to 97.929%. Similarly, in the backward strategy, the accuracy achieved using FS-4-ICIAR-BW has increased to 97.071% instead of 96.493% obtained in the first fusion stage.

The evaluation metrics for the second fusion stage accomplished with ICIAR 2018 dataset are described in Table 10. A sensitivity of 0.9809, a specificity equals to 0.9762, a precision equals to 0.988, and an F1-score equals to 0.9844 are obtained using FS-6-ICIAR-FW (forward strategy) On the other hand, for the backward strategy, FS-4-ICIAR-BW achieves a sensitivity equals to 0.9809, a specificity equals to 0.9524, a precision equals to 0.9739, and an F1-score equals to 0.9774.

**Table 10:** Performance metrics for sequential forward and backward search stratigies of the second fusion stage using MCS classifiers for the ICIAR 2018 dataset.

Classifier	Sensitivity	Specificity	Precision	F1-Score
**Forward strategy (FS-6-ICIAR-FW)**				
**MCS**	0.9809	0.9762	0.988	0.9844
**Backward strategy (FS-4-ICIAR-BW)**				
**MCS**	0.9809	0.9524	0.9739	0.9774

### Comparison with related studies

A comparison study is performed between Histo-CADx and the related works that used the exact datasets to measure its effectiveness as shown in Tables 11 and 12. Regarding the BreakHis dataset, it is clear from Table 12 that the accuracy of Histo-CADx is higher than Nahid & Kong (2018), Sudharshan et al. (2019), Jiang et al. (2019), Muralikrishnan & Anitha (2019), Alom et al. (2019), Vo, Nguyen & Lee (2019), Zhang et al. (2019), Li et al. (2020), and Toğaçar et al. (2020) for all magnification factors of BreakHis dataset except for the 200× where Histo-CADx has a slightly lower accuracy than Toğaçar et al. (2020). Regarding the ICIAR dataset, Table 13 verifies the competence of Histo-CADx compared to other recent related studies. The accuracy achieved by Histo-CADx is higher than Mahbod et al. (2018), Rakhlin et al. (2018), Nazeri, Aminpour & Ebrahimi (2018), Kausar et al. (2019), Roy et al. (2019), Sheikh, Lee & Cho (2020), Wang et al. (2020), and Brancati, Frucci & Riccio (2018). This is because some of the previous techniques used one to three CNNs networks individually to construct their CADx. Others studied the fusion of two or three CNNs, but no study has investigated the fusion of more than three CNN with handcrafted features. Moreover, earlier studies did not search for the best mixture of features that influence the performance of the CADx. Also, most of the previous studies did not take into consideration the high computational cost. Histo-CADx fused spatial features extracted from five CNNs along with two handcrafted feature extraction approaches using the AE DL method to consider the computational cost problem. It also examined the best combination of features that positively impacted the performance of the CADx. Additionally, it constructed a second stage of fusion to further improve the performance of CADx by developing a MCS which combined the outputs of three classifiers.

**Table 11:** A comparsion of recent related studies based on the BreakHis dataset.

Article	Accuracy (%)			
	Magnification Factor			
	40×	100×	200×	400×
(Nahid & Kong, 2018)	94.40	95.93	94.94	96.00
(Sudharshan et al., 2019)	87.8	85.6	80.8	82.9
(Jiang et al., 2019)	94.43	94.45	92.27	91.15
(Muralikrishnan & Anitha, 2019)	94.6	93.4	92.0	90.3
(Alom et al., 2019)	97.9	97.5	97.3	97.4
(Vo, Nguyen & Lee, 2019)	95.1	96.3	96.9	93.8
(Zhang et al., 2019)	91.2	91.7	92.6	88.9
(Li et al., 2020)	94.8	94.0	93.8	90.7
(Toğaçar et al., 2020)	97.99	97.84	98.51	95.88
Proposed Histo-CADx	99.03	99.53	98.08	97.56

**Table 12:** *A* Comparision with related studies based on ICIAR 2018 dataset.

Article	Accuracy (%)
(Mahbod et al., 2018)	88.50
(Rakhlin et al., 2018)	93.8
(Brancati, Frucci & Riccio, 2018)	97.3
(Nazeri, Aminpour & Ebrahimi, 2018)	95
(Kausar et al., 2019)	91
(Roy et al., 2019)	92.5
(Sheikh, Lee & Cho, 2020)	83
(Wang et al., 2020)	97.70
**Proposed Histo-CADx**	**97.93**

**Table 13:** The *s*ize of features and execution time before and after the fusion using AE.

Fusion	Size of features	Execution time
**Forward strategy (FS-5-BH-FW) BreakHis Dataset**		
**Before AE**	23,128	9 h 4 m 10 s
**After AE**	6,194	5 h 3 m 18 s
**Backward strategy (FS-1-BH-FW) BreakHis Dataset**		
**Before AE**	3,0229	12 h 9 m 50 s
**After AE**	9,562	7 h 19 m 24 s
**Forward strategy (FS-6-ICIAR-FW) ICIAR-2018 Dataset**		
**Before AE**	25,229	10 h 18 m 37 s
**After AE**	7,924	5 h 55 m 29 s
**Backward strategy (FS-4-ICIAR-BW) ICIAR-2018 Dataset**		
**Before AE**	20,642	7 h 51 m 5 s
**After AE**	5,348	3 h 47 m 48 s

## Discussion

BC is a severe malignant tumor that affects women all over the globe. Lately, it is considered one of the major causes of woman death (Alom et al., 2019). Detection of BC in its initial stage is essential to lessen the number of deaths among women. Regular investigations and screening are vital to distinguish between benign and malignant lesions. Several methods are used for imaging the BC, but the pathological analysis is favored due to its high ability to deliver direct evidence for the diagnosis, evaluation, and investigational treatments. Nevertheless, such analysis is complicated, wastes time, and labor-intensive. Moreover, even skilled pathologists might have different opinions about the case (Zhang et al., 2019). Thus, there is an essential need for an automated diagnostic tool that can assist a pathologist in their decision-making process and address such limitations.

**Figure 16:**
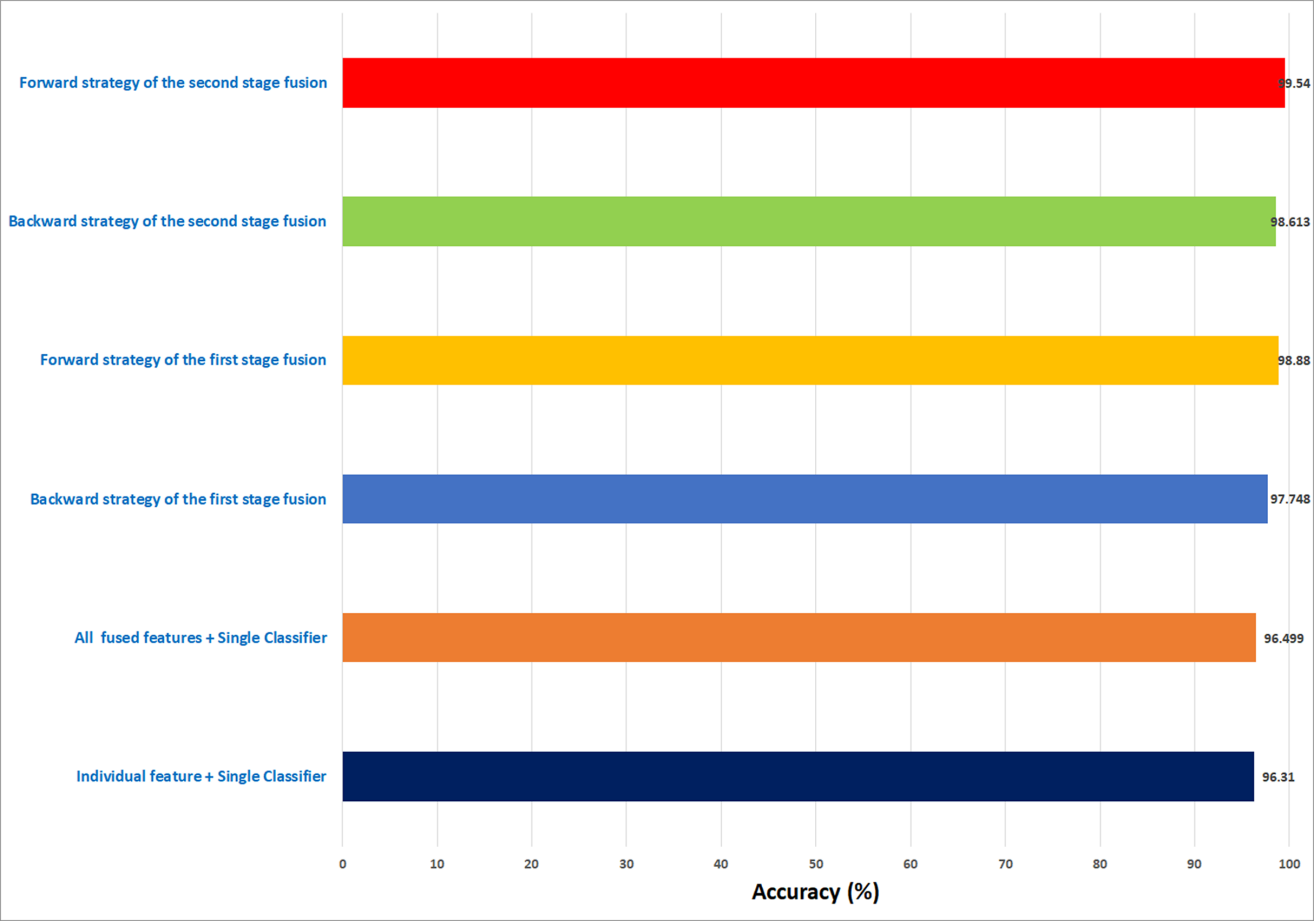
A summary of the highest accuracies achieved in the three experiments of the first stage fusion and the second stage fusion for the BreakHis dataset.

In this study, an automatic CADx system based on two cascaded fusion stages is proposed to aid pathologists in diagnosing BC. The first fusion stage showed that fusing multiple CNNs and handcrafted features increased the accuracy of Histo-CADx compared to CADx based on individual classifiers. Moreover, the sequential forward and backward strategies that have been used in the first stage fusion have further improved the accuracy of Histo-CADx. Additional improvements are achieved with the second fusion stage of Histo-CADx. As the accuracy of Histo-CAD using the MCS constructed using sequential forward and backward strategy has increased the accuracy to reach 99. 54% and 98.613% using the feature set FS-5-BH-FW 100× and FS-7-BH-BW 40× for the BreakHis dataset respectively as shown in Fig. 16.

**Figure 17:**
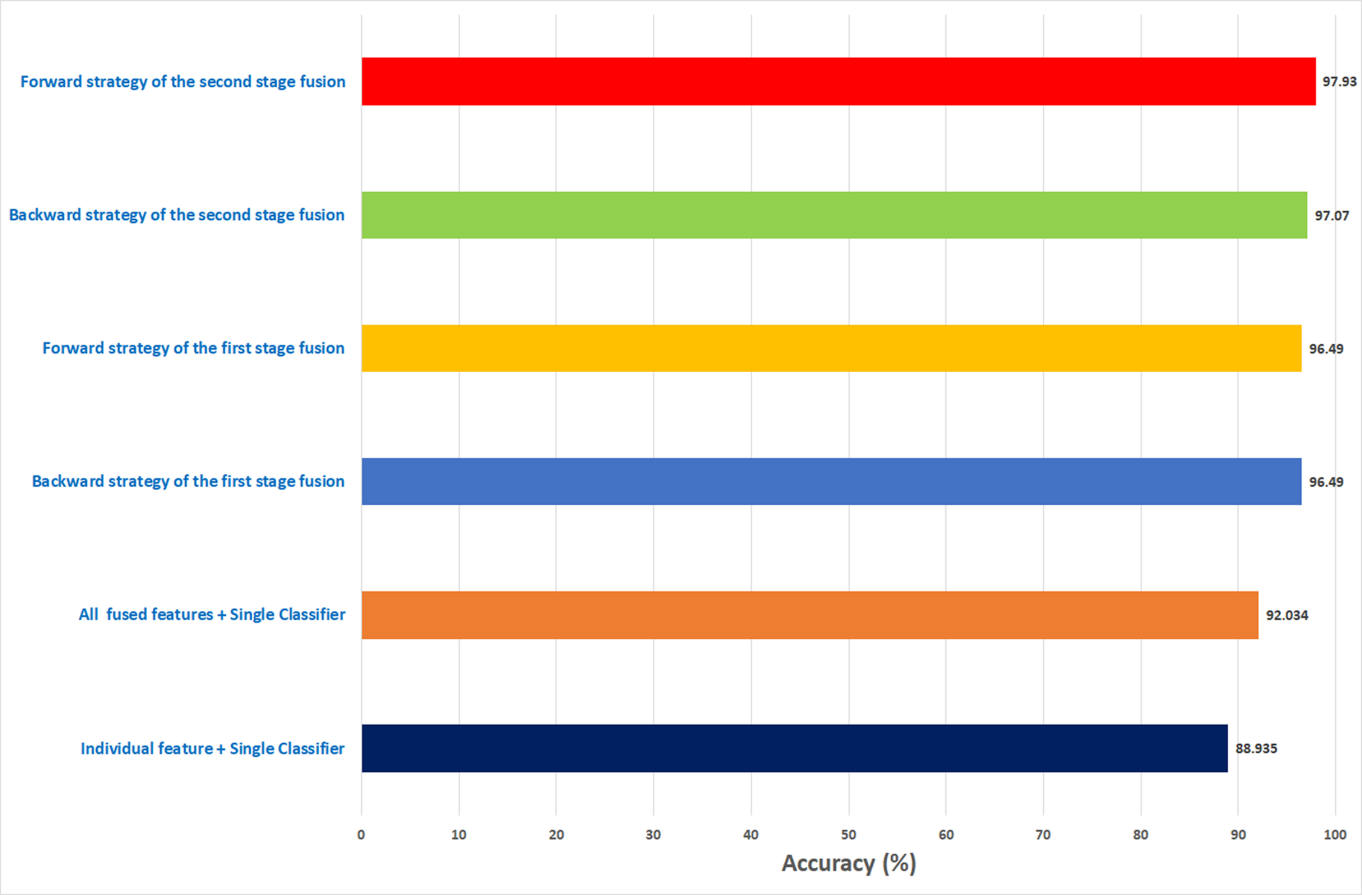
A summary of the highest accuracies achieved in the three experiments of the first stage fusion and the second stage fusion for the ICIAR 2018 dataset.

Similarly, the first fusion stage revealed that fusing multiple CNNs and texture feature extraction approaches have enhanced the classification ability of Histo-CADx. As proven in Hasan et al. (2020), Wei et al. (2019), Hansley, Segundo & Sarkar (2018), merging spatial features extracted from CNNs and traditional feature extraction techniques can boost the classification accuracy. Moreover, the sequential forward and backward strategies that have been utilized in the first stage fusion have additional improvement on the accuracy of Histo-CADx. This stage searched for the best combination of DL features that influence the classification accuracy of the system instead of combining all DL (Mehra, 2018; Nahid & Kong, 2018), which may lead to better performance. Further enhancements are obtained with the second fusion stage of Histo-CADx. As the accuracy of Histo-CAD obtained with the MCS using the sequential forward strategy has increased to reach 97. 93% and 97.07% using the feature set FS-6-ICIAR-FW and FS-4-ICIAR-BW for ICIAR 2018 dataset respectively as shown in Fig. 17. This is because the MCS usually enhances the classification accuracy (Attallah et al., 2017b; Krawczyk & Schaefer, 2014; Fusco et al., 2017; Fusco et al., 2012; Melekoodappattu & Subbian, 2020). Moreover, the fusion with the AE and MCS in the second fusion stage reduced both the fused feature dimension and the execution time for classification for both datasets as shown in Table 10. This is because the AE is a powerful feature reduction technique based on deep learning that can reduce the huge dimension of fused features which accordingly lowers the training time of the classification model (Xie et al., 2019; Chen et al., 2019).

It was previously described by Xie et al. (2019), Attallah (2020) that for a reliable classification model, the sensitivity should be more than or equal to 80%, specificity more than or equal to 95%, precision more than or equal to 95%. The sensitivities, specificities, and precision are shown in Tables 9 and 10 are all greater than 95%, therefore Histo-CADx can be considered a reliable system and can be used by pathologists to aid them and facilitate the diagnosis process.

## Conclusion

In this study, we presented Histo-CADx, a CADx system for the automatic classification of BC from histopathological images. Histo-CADx merges several spatial deep features and handcrafted feature extraction methods using the AE and MCS. The system consists of two cascaded fusion stages. The first stage consists of feature fusion which investigates and searches for the finest group of fused features which can enhance the performance of Histo-CADx. The second fusion stage depends on the fusion of three classifiers. The outputs of the three classifiers are fused using majority voting. The results of the first stage of fusion of Histo-CADx showed that the selected fused feature sets improved the accuracy of the CADx compared to CADx constructed with individual features. Moreover, the results of the second stage of fusion showed that this stage had further improvements on the performance of Histo-CADx. These results verified that Histo-CADx was capable of classifying BC more accurately compared to other recent studies, thus, it is a competitive system. It is also reliable, therefore, Histo-CADx may be used by doctors in attaining precise diagnosis. Moreover, it can decrease the time and hard work needed while examining the BC cases. It can be concluded from the results that combining multiple techniques in stages and fusing the best outputs of the stages provided an increase in the performance of the system. The results of the proposed Histo-CADx showed that using techniques applied in stages and using more CNNs can out-perform systems with fewer stages and CNNs. This may suggest further improvements to be made with more stages. Forthcoming work will consider exploring further CNNs and handcrafted approaches. Further analysis regarding the use of handcrafted feature extraction methods will be made. Besides, the influence of employing them on the result will be investigated. Furthermore, a multi-center study can be carried out to assess the performance of the proposed CADx system and ensure the portability of the solutions presented. Future experiments can also be conducted to extend the model to even aid in identifying different types of malignancy.

## Supplemental Information

10.7717/peerj-cs.493/supp-1 Supplemental Information 1 Codes.

10.7717/peerj-cs.493/supp-2 Supplemental Information 2 Samples for images available in the datasets.

## Additional Information and Declarations

### Data Availability

The following information was supplied regarding data availability: The BreakHis dataset is available at: Spanhol, F., Oliveira, L. S., Petitjean, C., Heutte, L., A Dataset for Breast Cancer Histopathological Image Classification, IEEE Transactions on Biomedical Engineering (TBME), 63(7):1455-1462, 2016. https://web.inf.ufpr.br/vri/databases/breast-cancer-histopathological-database-breakhis/. The database requires interested parties to register to request access. The ICIAR 2018 dataset is available at: https://iciar2018-challenge.grand-challenge.org/dataset/, DOI 10.1016/j.media.2019.05.010. The organizers require interested parties to register to request access to the dataset. Codes are available in the Supplemental File.
